# Jinshui Chenfei formula alleviates SiO_2_-induced pulmonary fibrosis by inhibiting macrophage M2 polarization via Grb2/STAT6 in rats

**DOI:** 10.1186/s13020-026-01339-7

**Published:** 2026-03-16

**Authors:** Fan Yang, Runsu Hou, Xinguang Liu, Yan Du, Qin Zhang, Xin Pan, Peng Zhao, Yange Tian, Jiansheng Li

**Affiliations:** 1 Henan Key Laboratory of Chinese Medicine for Respiratory Disease, Henan University of Chinese Medicine, 156 Jinshui Dong Road, Zhengzhou, 450046 Henan Province China; 2 Collaborative Innovation Center for Chinese Medicine and Respiratory Diseases co-constructed by Henan Province & Education Ministry of P.R. China, Zhengzhou, 450046 Henan Province, China; 3 Academy of Chinese Medical Sciences, Henan University of Chinese Medicine, Zhengzhou, 450046 Henan Province China; 4 Department of Respiratory Diseases, the First Affiliated Hospital of Henan University of Chinese Medicine, Zhengzhou, 450000 Henan Province, China

**Keywords:** Jinshui Chenfei formula, Silicosis, Macrophages of M2 polarization, STAT6, Grb2

## Abstract

**Background:**

Jinshui Chenfei Formula (JCF), a proprietary Chinese medicinal prescription, has demonstrated remarkable therapeutic efficacy in treating pneumoconiosis patients. However, the precise mechanisms remain to be elucidated. This study aimed to investigate how JCF counteracts M2 macrophage polarization and thereby attenuates SiO_2_-triggered silicosis progression.

**Methods:**

This study aimed to observe the effects of JCF on collagen deposition and macrophage M2 polarization in silicotic lungs induced by silica at 14 days, 28 days, and 42 days in rats. The active fraction of JCF was isolated and extracted using macroporous resin. Subsequently, RNA-seq was performed to predict the potential mechanism involved in the improvement of IL4-induced bone marrow-derived macrophages (BMDMs) by JCF. Finally, the targets of the active fraction of JCF were identified by the DARTS experiment. The underlying mechanisms were elucidated by combining siRNA and plasmid overexpression techniques.

**Results:**

JCF significantly improved pulmonary function and pathological remodeling in silicosis rats across different time points, reduced collagen deposition in lung tissues, and downregulated the expression of M2 macrophage markers. JCF5, the active fraction of JCF, potently inhibited IL4-induced M2 polarization of bone marrow-derived macrophages (BMDMs). RNA-seq identified 96 differential expressed genes (DEGs) associated with M2 polarization and JCF5 treatment, which enriched signaling pathways such as JAK/STAT. In vitro experiment confirmed that JCF5 markedly reduced STAT6 phosphorylation. Drug affinity responsive target stability (DARTS) assays revealed that JCF5 suppress STAT6 activation by down-regulating Grb2, siRNA-mediated Grb2 knockdown potently reduced IL-4-induced M2 macrophages, whereas Grb2 overexpression significantly alleviated the inhibitory effects of JCF5 on STAT6 activation and M2 polarization. Further, Hesperidin is the main active ingredient verified by molecular docking and cell function assays.

**Conclusion:**

JCF downregulate STAT6 activation by regulating Grb2, thereby inhibit M2 polarization of macrophage and improve pulmonary fibrosis in silicotic rats.

**Supplementary Information:**

The online version contains supplementary material available at 10.1186/s13020-026-01339-7.

## Introduction

Silicosis is an irreversible occupational interstitial lung disease caused by long-term work or living in a silicon dust environment. It is characterized by persistent inflammation and permanent pulmonary fibrosis and can be divided into acute, accelerated, and chronic stages [12, 19]. From 1990 to 2019, the incident and prevalent cases of silicosis increased by 64.6% and 91.4%, respectively [17]. At present, the only existing drug, tetrandrine, approved for silicosis in China, can alleviate the clinical symptoms and pulmonary function of silicosis patients [23], but it cannot block or reverse the progression of the disease and exhibits side effects including sodium deficiency, bloating, and liver injury [6]. Therefore, the development of safe and effective treatment agents is urgent.

Silicon dioxide (SiO_2_) is the main silicon dust that enters lung tissue with respiration, which activates various effector cells in lung tissues, in which the vicious cycle of SiO_2_ swallowed by alveolar macrophages (AMs) and their release induces inflammation, granulomatous nodules, and irreversible fibrogenesis [1]. When SiO_2_ was identified and activated by AMs, the macrophage phenotype shifted from M0 to the activation state [3]. According to their surface markers and function, macrophages can be classified as classically activated macrophages (M1) or alternatively activated macrophages (M2) [25]. In the acute stage, M1 macrophages were induced to kill pathogens and promote inflammatory injury by releasing large amounts of cytokines such as tumor necrosis factor α (TNF-α), interleukin-1β (IL-1β) and IL-6. In the middle to late stage, M2 macrophages were stimulated and exerted an effect on anti-inflammatory and repairing damaged tissue through secreting transforming growth factor-β (TGF-β) and other cytokines to induce the proliferation and differentiation of fibroblasts, accelerating the collagen deposition [24, 26]. Chronic macrophage polarization can trigger excessive tissue inflammation and fibrogenesis [11]. Therefore, inhibition of M2 macrophage polarization is a potential measure to ameliorate pulmonary fibrosis.

Recently, traditional Chinese medicine (TCM) has shown good safety and efficacy in treating silicosis [22]. Jinshui Chenfei formula (JCF) is a novel prescription used to treat pneumoconiosis patients at all stages. Its clinical feasibility has been demonstrated using multicenter randomized controlled trials, which showed significantly improving patient symptoms and lung function, increasing six-minute walking distance and exercise endurance, and improving quality of life [15]. In this study, a silicosis rat model was established to explore how JCF affects M2 polarization of macrophages and alleviates silicosis fibrosis through intermediate pathways.

## Materials and methods

### Materials and reagents

Silicon dioxide (SiO2) particles, with a size distribution ranging from 0.5 to 10 μm, were provided by Sigma Co. LLC. (Louis, MO, USA). Tetrandrine (20 mg × 36 pills/box, PH2003006) was purchased from CONBA Bio-pharm Co., Ltd. (Zhejiang, China).

### Animals and treatments

Ninety-six male Sprague–Dawley rats (6–8 weeks) were provided by Vital River Co., Ltd. (Beijing, China). All animals lived in a pathogen-free facility and were given feedstuff and water ad libitum. All animal experiments were approved by the Ethics Committee for Animal Welfare at Henan University of Chinese Medicine (Zhengzhou, China) (DWLL202103141).

**Fig. 1:**
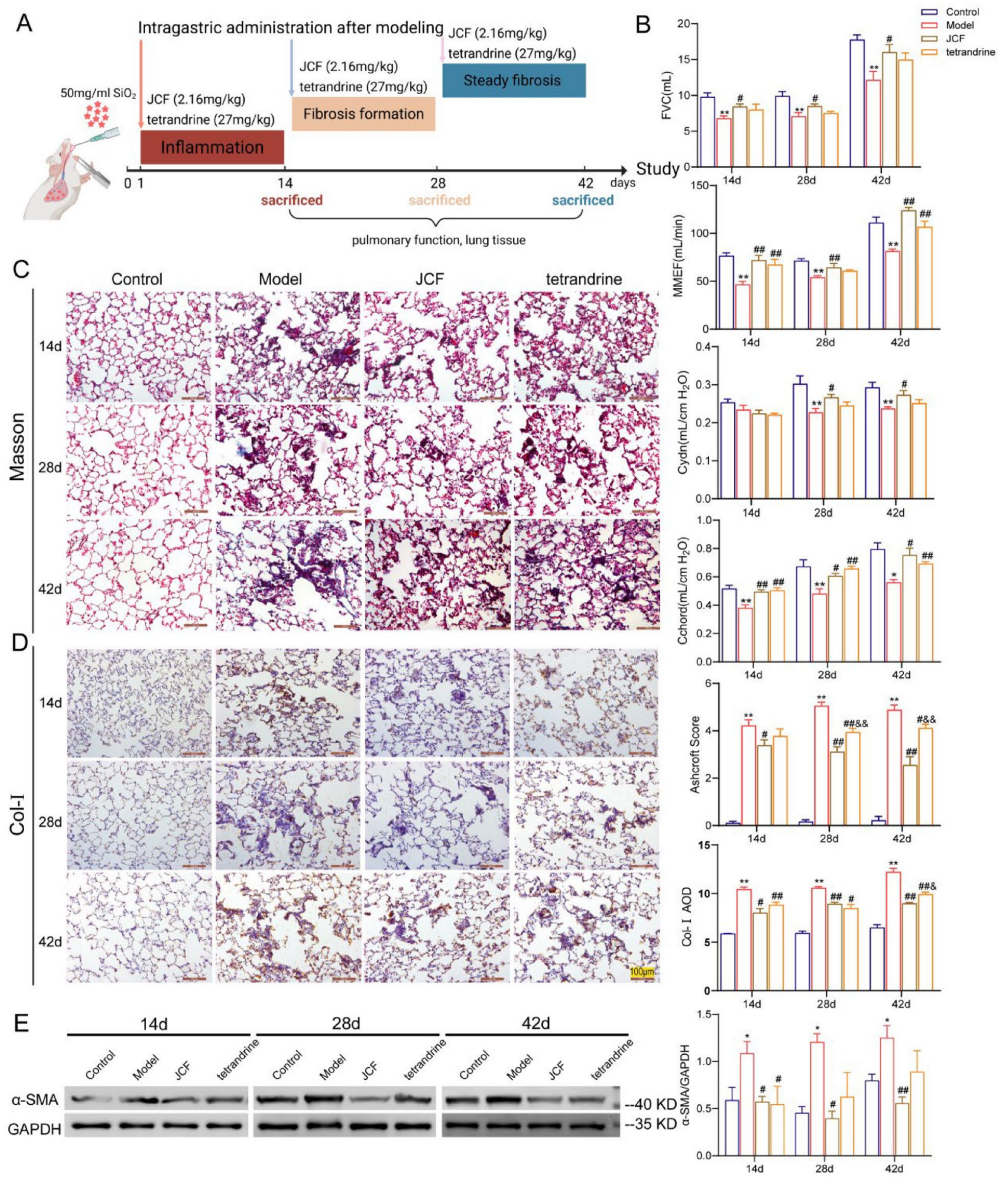
Jinshui Chenfei formula (JCF) improves lung function and tissue pathology in silicotic rats. **A** Schematic diagram of the silicosis model and drug intervention; **B** Changes of the forced vital capacity (FVC), maximal mid expiratory flow (MMEF), Dynamic lung compliance (Cydn), and static lung compliance (Cchord); **C** Representative images of Masson trichrome staining and fibrosis scores in lung tissue at 200 × magnification; **D** Representative images of the expression of Col-I, and the average optical density in lung tissue at 200 × magnification; Scale bar = 100 μm; **E** The protein level of α-SMA detected by Western blot. (*P < 0.05, **P < 0.01, vs. the Control; ^#^*P* < 0.05, ^##^*P* < 0.01, vs. the Model; ^&^*P* < 0.05, ^&&^*P* < 0.01, vs. the JCF group)

After one week of adaptive feeding for all animals, the silicosis model was established using one-time non-exposed intratracheal instillation of silica suspension. After abdominal anesthesia, 1 ml of SiO_2_ suspension (50 mg/ml) dissolved with normal saline was instilled intratracheally in the model group, and the same volume of normal saline was instilled in the control group. The 3 phases of the 14-day administration treatment started on days 1, 15, and 29, respectively. The JCF extract (2.16 g/kg) and tetrandrine (27 mg/kg) were given in the JCF group and tetrandrine group, respectively. And the control group and model groups received distilled distilled water. All animals were sacrificed at 14, 28, and 42 days, separately (Fig. 1A).

### Preparation of JCF extract

The drugs in the JCF were purchased from Ruilong Pharmaceutical Co. (Henan, China). All herbs were decocted and filtrated twice using 12 times water (1 h each time). And the supernatant were collected after filtering the decoction and combined into a concentrated solution and baked into powder using an electronic oven. And 1 g of powder contains 4.51 g of Chinese herbs. This powder was dissolved in distilled water and administered to silicosis rats.

The JCF powder was loaded onto a DM101 macroporous-resin column and step-eluted with 0%, 10%, 30%, 60%, 90% and 100% aqueous ethanol. The six corresponding effluents were collected, concentrated to dryness and labelled JCF1-6, respectively. Each dry extract was dissolved in dimethyl sulfoxide (DMSO) at 50 mg/ml solution and stored at −20 ℃ for further experiments in vitro.

### Pulmonary function test

At 14, 28, and 42 days following the establishment of the silicosis model, the forced vital capacity (FVC), maximal mid-expiratory flow curve (MMEF), dynamic lung compliance (Cdyn) and static lung compliance (Cchord) was assessed using PFT testing system (DSI, St. Paul, MN, United States) after the rats had been anesthetized.

### Histomorphology

After being fixed in 4% paraformaldehyde solution, the left lung tissues specimens of rats were embedded, sectioned, and stained with hematoxylin–eosin (HE) solution (Solarbio, Beijing, China), and Masson stain kit (Solarbio). The degree of inflammation and fibrosis was assessed by alveolitis scoring (Szapiel et al. 1979)^27^ and Ashcroft scoring (Ashcroft et al. 1988)^28^.

### Immunohistochemistry

After deparaffinization, antigen retrieval, and blocking, the lung slices were incubated with primary antibodies overnight at 4 ℃, including Col-Ⅰ, α-SMA, CD206, and Arg1. After that, they were incubated with secondary antibodies at room temperature and stained using diaminobenzidine (DAB) solution (Servicebio, Wuhan, China). Finally, the integrated optical density (IOD) of their positive staining area was analyzed using Image-Pro Plus 6.0.

### Cell culture and treatment

Bone marrow-derived macrophages (BMDMs) were extracted from the femur and tibia of both hind limbs of C57BL/6J mice (6–8 weeks) using PBS with 4% fetal bovine serum (FBS). After lysing and washing the red blood cells, the remaining cells were plated in 6-hole dishes with RPMI 1640 (Solarbio, Beijing, China) supplemented with 20 ng/ml of mouse Macrophage colony-stimulating factor (M-CSF) (Yeasen, Shanghai, China) cultured for 7 days. Next, BMDMs were treated with 50 μg/ml JCF1-6 and JCF5 (50, 25 and 12.5 μg/ml) for 3 h, followed by stimulation with 20 ng/ml IL-4 for 48 h. Finally, samples were collected.

### Western blot analysis

The sample of right lung tissue and cells were lysed with Ripa lysis buffer, and their concentration determined using BCA protein concentration assay kit (YAMAY, Shanghai, China). Proteins were denatured in a 100 ℃ metal bath for 10 min, resolved on 10% SDS polyacrylamide gels and imprinted onto PVDF membranes. After blocking, membranes were incubated with primary antibodies overnight at 4 ℃, including CD206, Arg-1, α-SMA, p-STAT6, STAT6, Grb2 and GAPDH. Following incubation with HRP-conjugated secondary antibodies, bands were visualized with an imaging system (Bio-Rad, USA) and quantified by ImageJ.

### Real-time PCR

Total RNA of cells was extracted using RNA extraction solution (Servicebio, Wuhan, China), freverse-transcribed with HiScript II Q RT SuperMix (Vazyme, Nanjing, China). qPCR was then performed on an Applied Biosystems QuantStudio 6 (Thermo Fisher, CA, USA) using ChamQ Universal SYBR qPCR Master Mix (Vazyme). The gene-specific primer sequences are listed in Table 1.

**Table 1:** Primer information

Gene name		Primer sequence(5’−3’)
GAPDH	Forward	AGGTCGGTGTGAACGGATTTG
	Reverse	GGGGTCGTTGATGGCAACA
CD206	Forward	AAGGCATGCGTTGCACATAC
	Reverse	ATTCTGCTCGATGTTGCCCA
Arg-1	Forward	TGTCCCTAATGACAGCTCCTT
	Reverse	GCATCCACCCAAATGACACAT
TGF-β1	Forward	CTCCCGTGGCTTCTAGTGC
	Reverse	GCCTTAGTTTGGACAGGATCTG
PDGF-AA	Forward	TGGCTCGAAGTCAGATCCACA
	Reverse	TTCTCGGGCACATGGTTAATG
Grb2	Forward	GAAGTATTTCCTGTGGGTGGTGAAG
	Reverse	GCTGTGGCATCTGTTCTATGTCC

### RNA sequencing

The total RNA of BMDMs cells were extracted with TRIZOL regents (Invitrogen, Carlsbad, CA, USA) following by manufacturer’s protocol. Differently expressed genes among the Control, IL-4, and JCF5 groups were identified by RNA sequencing (Jingzhou Gene Technology Co., Ltd. Shanghai, China). The GO functional enrichment (biological process, cellular component, molecular function) and KEGG pathway analysis of JCF5-reversed genes were performed with Metascape database (https://metascape.org) and visualized on the Bioinformatics platform (http://www.bioinformatics.com.cn).

### Drug affinity responsive target stability (DARTS)

DARTS is a commonly technique used for discovering small molecule targets of drugs. BMDMs were collected and lysed in M-PER mammalian protein extraction reagent (sigma, Louis, MO, USA) and the lysate were diluted with TNC buffer [1 M Tris–HCl (pH 8.0), 5 M NaCl, 1 M CaCl2]. After splitting into two equal portions, the lysate was incubated with JCF5 (50 μg/ml) or vehicle (DMSO) for 2 h. Each portion was then divided into four aliquots and subjected to limited proteolysis with pronase at 0, 1:400, 1:1,000, or 1:2,000 dilutions for 2 min. The reactions were stopped by adding loading buffer and heating. Proteins were resolved on 10% SDS-PAGE gels, stained with Coomassie Brilliant Blue for 2 h, and imaged with a ChemiDoc system (BIO-RAD, USA).

The gels with significant trend between DMSO and JCF5 were collected, followed treated with decolorization, dehydration and enzymolysis, the supernatants were extracted and dried, which were dissolved in 0.1% formic acid for LC–MS/MS identification. The protein–protein interaction (PPI) of these targets were analyzed using STRING database (https://string-db.org).

### Cell transfection

BMDMs were inoculated on 6-well dishes for cell transfection. (1) The siRNA or plasmid solution and Lipo 6000^™^ (Beyotime, Shanghai, China) were mixed with Opti-MEM (Gibco, CA, USA) for 5 min according to the experimental protocol. (2) The transfection mixture was added into BMDMs, which were gently shaken and incubated for 4–6 h. (3) After incubation, the culture medium containing the transfection mixture was replaced with fresh medium and incubation continued for 18–20 h for subsequent experiments.

### Immunofluorescence

The BMDMs samples were blocked with goat serum, incubated with first antibody and then with the corresponding fluorescence-conjugated secondary antibody. Finally, the samples images were detected by fluorescence microscopy (OLYMPUS FV1000, Japan). And images were analyzed using Image J software.

### Molecular docking

The protein sequence and PDB format file of Grb2 (Uniprot ID: Q60631) were obtained from Uniprot database (https://www.uniprot.org/) and the Swissmodel database (https://swissmodel.expasy.org/interactive). The 2D structured SDF format files of 5 critical ingredients, Licorice glycoside C2, Zhebeirine, Eriocitrin, Hesperidin and Melitidin, were downloaded from the PubChem database (https://pubchem.ncbi.nlm.nih.gov/). These SDF format were converted into mol2 format files using Open Babel GUI software. The PyMOL software was used to preprocess before docking and finally export the pdbqt format file. Finally, it was processed and docking analysis was performed using AutoDock software.

### Statistical analysis

Data were exhibited as mean ± SEM. Which were analyzed by unpaired *t-test* for two groups comparisons and *one-way ANOVA analysis* for multiple groups; *P* < 0.05 was defined as statistical significance.

## Result

### JCF improves lung function and tissue pathology in silicotic rats

In our previous studies in rats with the silicosis model, we found a progressive worsening of lung histopathology after SiO_2_ exposure, which manifested as an inflammatory phase of 0–14 days, a fibrotic formation phase of 15–28 days, and a steady fibrotic phase of 29–42 days. To evaluate the effect of JCF and tetrandrine administration on silicosis at different pathological stages, rats were separately administrated JCF and tetrandrine by intragastric at 1 day, 15 days, and 29 days for 2 weeks after one-off non-invasive instillation of SiO_2_ (Fig. 1A). From pulmonary function analysis, FVC, MMEF, Cdyn, and Cchord were significantly decreased in the model group at 3 pathological stages, and JCF and tetrandrine administration can significantly reverse these changes (Fig. 1B). The inflammatory cells infiltration and damage to alveolar structure around SiO_2_ particle were observed in the model group on day 14. As the disease progressed, fibrous cords and silicotic nodules appeared on day 28, while more severe lesion ranges and the degree of fibrosis appeared on day 42. These alterations were ameliorated after treatment with JCF and tetrandrine. In addition, we assessed the degree of pulmonary pathological damage by scoring alveolar inflammation and fibrosis, which showed that alveolitis and fibrosis scores were increased compared to that of the control group, whereas these scores were significantly decreased in the rats treated with JCF and tetrandrine (Fig. 1C and Fig. S1). JCF and tetrandrine exhibit comparable pharmacological efficacy, with JCF demonstrating superior reduction in Ashcroft scores relative to tetrandrine on days 28 and 42.

Excessive extracellular matrix deposition is a key pathology in the progression of silicosis fibrosis, with collagen deposition being the most prominent. Here, we used immunohistochemistry (IHC) to detect the expression of collagen I (Col-Ⅰ) and α-smooth muscle actin (α-SMA) in lung tissues of rats with silicosis. As the SiO_2_ exposure time increases, collagen fibers around SiO_2_ in lung tissue of silicosis rats gradually increased, and the positive expressions of Col-I and α-SMA increased significantly, peaking on day 42. In addition, JCF and tetrandrine treatment reduce their level compared to the model group at each time point (Fig. 1D and Fig. S2). Meanwhile, the protein expression level of α-SMA in lung tissue homogenate was consistent with these findings (Fig. 1E). Meanwhile, JCF lowered Col-I and α-SMA expression more effectively than tetrandrine on day 42.

### JCF inhibits macrophages M2 polarization in silicotic rats

**Fig. 2:**
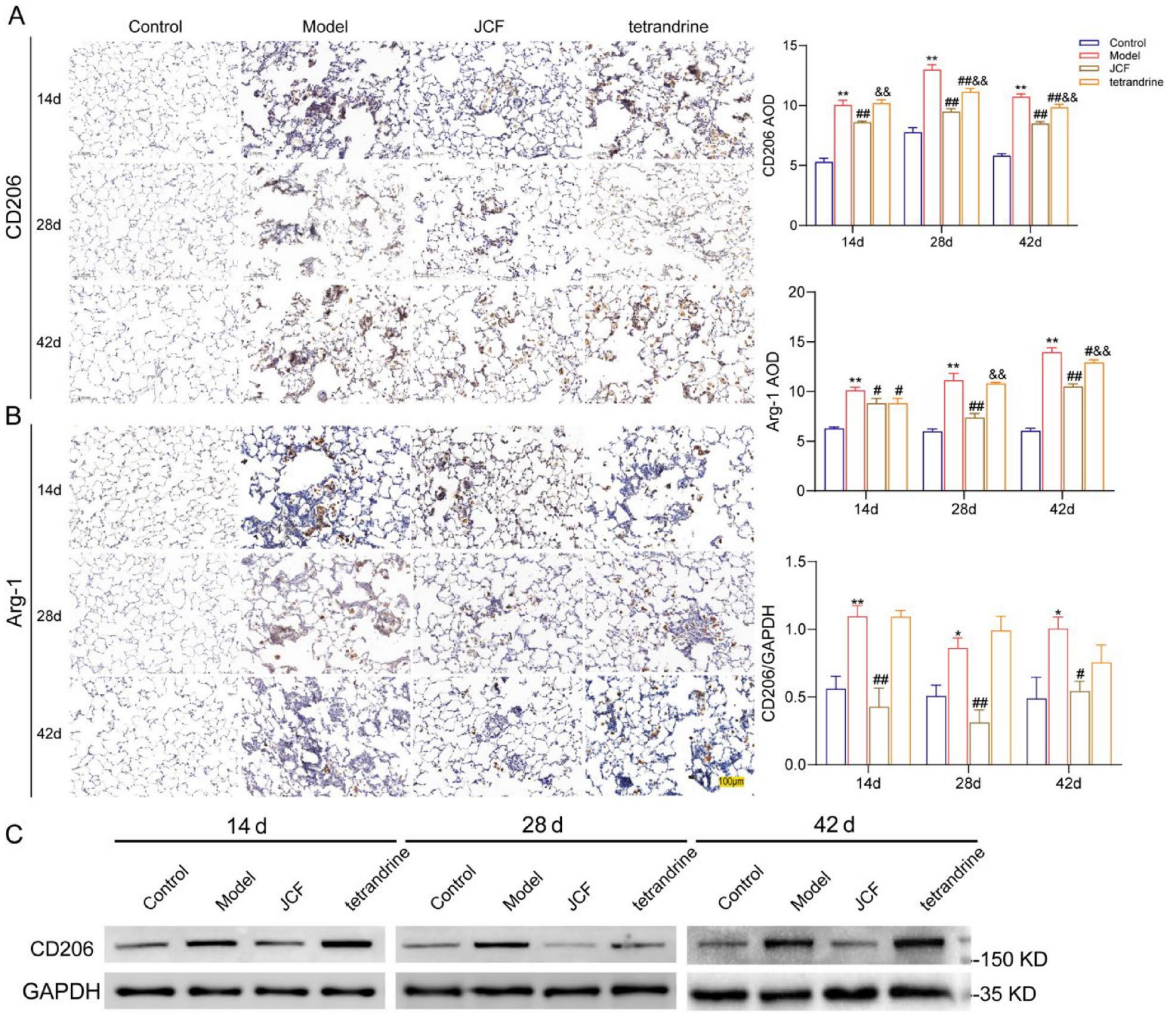
JCF decreases the transition of M2 macrophages in silicotic rats. **A** Representative images of the expression and the Average optical density of Arg-1 in lung tissue at 200 × magnification; **B** Representative images of the expression and the Average optical density of CD206 in lung tissue at 200 × magnification; Scale bar = 100 μm; **C** The western blot analysis of CD206 expression in lung homogenate of each group. (*P < 0.05, **P < 0.01, vs. the Control; ^#^*P* < 0.05, ^##^*P* < 0.01, vs. the Model; ^&^*P* < 0.05, ^&&^*P* < 0.01, vs. the JCF group)

Alternatively activated macrophages (M2 macrophages) are involved in tissue repair processes in the progression of pulmonary fibrosis (Zhang Z et al., 2022). To investigate the effect of JCF on the polarization of M2 macrophages in silicotic rats, we detected the level of M2 phenotype macrophage markers (CD206 and Arg-1) at different time points of SiO_2_ exposure using IHC. The results showed that M2 macrophages were mainly accumulated in the injury area of SiO_2_ and highly expressed in silicotic rats. In addition, compared with the control group, the expression of CD206 and Arg-1 gradually increased with the prolongation of silica exposure time in silicotic rats, peaking on day 42. Compared with the Model group, the expression of M2 macrophage markers was significantly reduced in the JCF and tetrandrine treatment (Fig. 2A, B). Meanwhile, we also detected the protein expression of CD206 in rat lung tissue; the protein expression level of CD206 was consistent with IHC findings (Fig. 2C). Meanwhile, JCF exhibited a greater reduction in Arg-1 and CD206 expression than tetrandrine on days 28 and 42. These findings indicated that JCF may delay the progression of silicosis and alleviate silicosis fibrosis by inhibiting M2 macrophages.

### JCF5 inhibits M2 polarization in bone marrow macrophages (BMDMs)

**Fig. 3:**
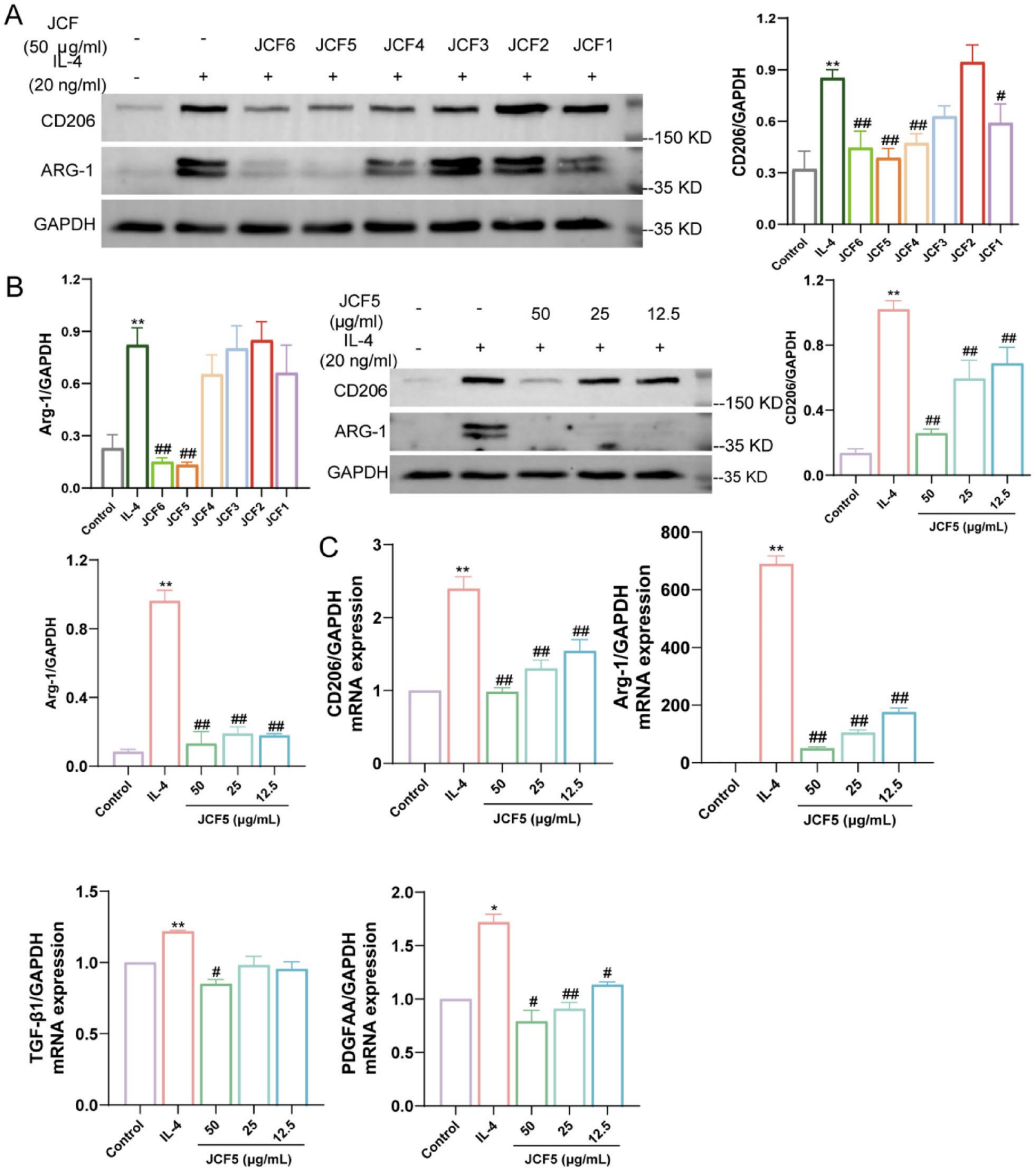
JCF5 inhibits M2 polarization in bone marrow macrophages (BMDMs) induced by IL-4. **A** The protein level of CD206 and Arg-1 in BMDM cells treated with JCF1-6. **B** The protein level of CD206 and Arg-1 treated with JCF5. **C** The mRNA expression level of CD206, Arg-1, TGF-β1, and PDGF-AA in BMDMs treated with JCF5. (*P < 0.05, **P < 0.01, vs. the Control; ^#^*P* < 0.05, ^##^*P* < 0.01, vs. the IL-4 group)

To investigate the effective substance and intervention mechanisms of JCF in vitro, JCF was purified and separated into six fractions (JCF1-6) by using macroporous resin. Subsequently, BMDMs were treated with JCF1-6 and interleukin-4 (IL-4). JCF5 and JCF6 were found to decrease the protein expression of M2 polarization markers (CD206, Arg1; Fig. 3A). JCF5 was used as the representative active ingredients of the Jinshui Chenfei formula for the following drug mechanism researches.

To investigate the therapeutic effect of JCF5 on BMDM cells induced by IL-4, the safe concentration range of JCF5 was determined using the CCK-8 assay (Fig. S3). Gradient JCF5 was used to treat BMDMs in 50, 25, and 12.5 μg/ml. The result showed that JCF5 reduced the protein level of CD206 and Arg-1 with dose-dependent (Fig. 3B). Meanwhile, the mRNA expressions of CD206, Arg-1, TGF-β1, and PDGF-AA were remarkably decreased (Fig. 3C). These results reveal that M2 macrophage polarization induced by IL-4 could be significantly inhibited by JCF5.

### JCF5 regulates JAK/STAT signaling pathway to inhibit macrophages M2 polarization

**Fig. 4:**
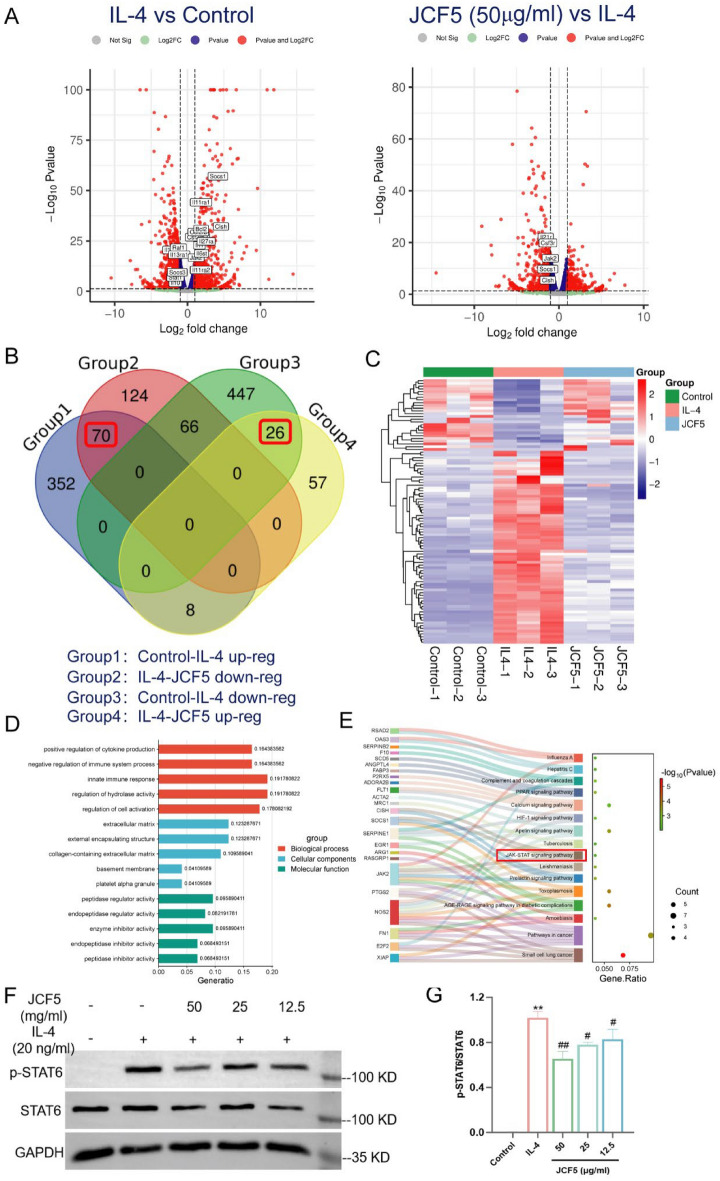
The DEGs analysis among Control, IL-4 and JCF5 groups. **A** Volcanic plot of differentially expressed gene expression in IL-4 vs. Control and JCF5 vs. IL-4. The red dots indicate up-related genes and the blue dots indicate down-related genes; **B** The venn diagram shows the number of differentially expressed genes and their overlap in up- and down-regulated genes of Control vs. IL-4 and IL-4 vs. JCF5 group. The numbers in the red box represent the differentially reversed genes, which up-regulated in Control vs. IL-4 and down-regulated in IL-4 vs. JCF5 group; **C** The heatmap of 96 differentially reversed genes in Control, IL-4 and JCF5 group; GO functional enrichment analysis (**D**) and the sankey dot pathway enrichment pathway (**E**) of 96 differentially reversed genes. **F**-**G** The ratio of phosphorylated STAT6 to total STAT6 in BMDM cells treated with JCF5. (*P < 0.05, **P < 0.01, vs. the Control; ^#^*P* < 0.05, ^##^*P* < 0.01, vs. the IL-4 group)

Transcriptomics quantifies changes in gene expression during treatment or disease states to analyze differentially expressed genes (DEGs), which can be used to develop and discover molecular features and biomarkers for diseases or effective drug effects [4]. 969 genes were identified to be regulated in the IL-4 group (compared to the control group), which contained 430 up-regulated and 539 down-regulated genes. In addition, we also found that 351 genes were detected in the 50 μg/ml JCF5 group (compared to the IL-4 group), which included 91 up-regulated and 260 down-regulated genes (Fig. 4A). According to further analysis, we identified 96 DEGs that JCF5 reverse IL-4-induced M2 macrophage polarization, and their expression in each group (Fig. 4B, C). The Kyoto Encyclopedia of Genes and Genomes (KEGG) pathway enrichment and GO functional analysis of the 96 reverse genes were enriched with 265 GO terms and 16 KEGG terms (Fig. 4D, E). As shown in multiple sets of bar charts, the top 5 terms of biological processes (BPs), cellular components (CCs), and molecular functions (MFs) categories were listed. And the BPs were associated with regulation of cytokine production and immune system processes. The CCs were enriched on extracellular matrix, platelet alpha granule, actin-based cell projection, etc. And then, the MFs were related to peptidase regulator activity, oxidoreductase activity, growth factor binding, etc. The 16 KEGG pathways were listed in bubble charts, which were mainly enriched in pathways in cancer, the calcium signaling pathway, the JAK/STAT signaling pathway, and so on. Importantly, the activation of STAT6 is a critical signal in M2 polarization of macrophages [25]. We found that JCF5 obviously decreased the protein expression of phosphorylation of STAT6 (Fig. 4F, G). The results suggested that JCF5 could restrain M2 polarization of macrophages through reducing the activation of STAT6.

### Grb2 is the target of JCF5 and promote macrophage M2 polarization

**Fig. 5:**
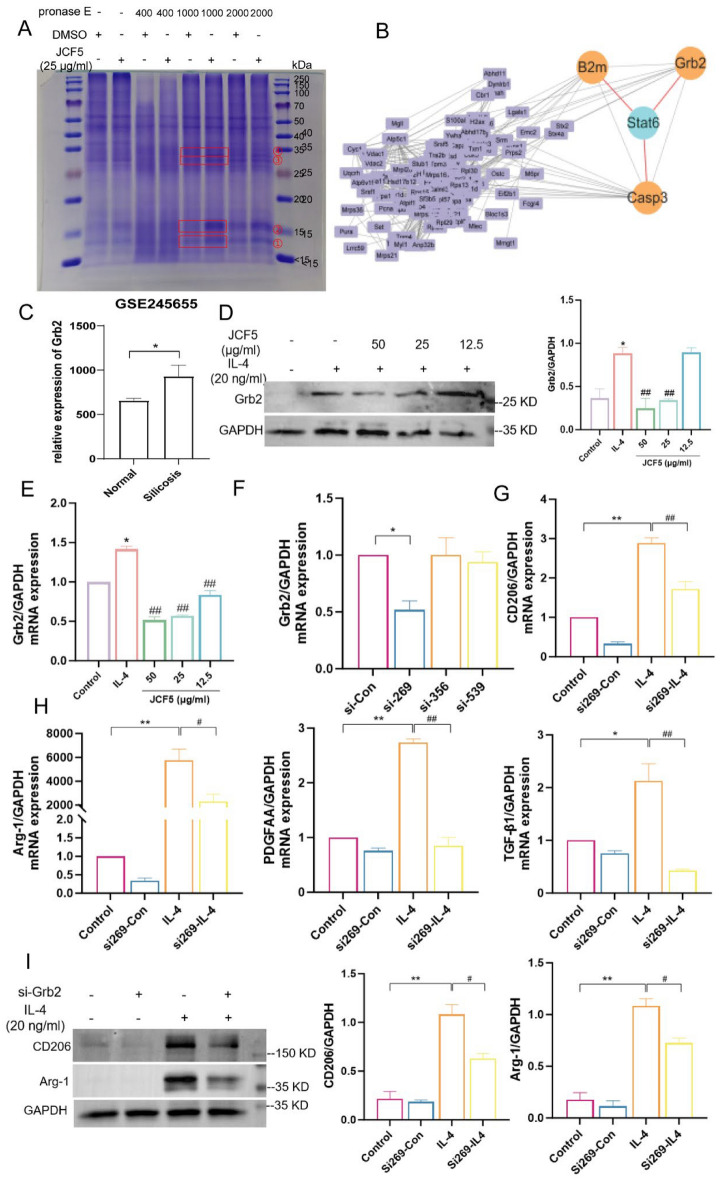
Grb2 regulates macrophages of M2 polarization. **A** The gels of bands in the control (DMSO) and the JCF5 group at different concentrations of pronase E. The red rectangle indicated the 4 differentially expressed bands. **B** PPI network of STAT6 and 151 targets in JCF5, which were obtained from differential bands. The orange oval shape indicated the interacted targets of STAT6. **C** The expression of Grb2 in the GSE245655 dataset. **D**, **E** The protein and mRNA level of Grb2 in BMDMs cells. **F**–**H** The mRNA expression of Grb2, CD206, Arg-1, PDGF and TGF-β1 in BMDMs cells. **I** The protein level of CD206 and Arg-1 in BMDM cells silenced by si-Grb2. (*P < 0.05, **P < 0.01, vs. the Control; ^#^*P* < 0.05, ^##^*P* < 0.01, vs. the IL-4 group)

The drug affinity responsive target stability (DARTS) coupled with mass spectrometry (MS) analysis was employed to identify the targets of JCF5 in BMDMs. Gel electrophoresis results revealed that JCF5 expression was significantly upregulated at 4 gel strip positions relative to DMSO (Fig. 5A). Subsequent MS analysis of these 4 gel strips resulted in the identification of 151 targets showing expression trends (Tab. S1 and Fig. S4). Upon importing the 151 targets and STAT6 into the STRING database, a high-confidence protein–protein interaction network was identified among STAT6, β2-microglobulin (B2M), caspase-3 (CASP3), and growth factor receptor-bound protein 2 (Grb2) (Fig. 5B). Grb2 functions as a signal amplifier in IL-4-induced M2 macrophage polarization by indirectly facilitating JAK-dependent STAT6 phosphorylation.

In addition, the GSE245655 database showed that the expression of Grb2 significantly increased in proteomics of lung tissue in silica-mediated silicosis mice (Fig. 5C). Our results indicated that the expression of Grb2 was remarkably decreased in BMDM cells treated with JCF5 (Fig. 5D, E). To evaluate the biological function of Grb2 in M2 macrophage polarization, small interfering (si)-RNAs against Grb2 were alternatively transfected into BMDM cells before treatment with IL-4. Three si-Grb2 sequences (si-Grb2 269, si-Grb2 356, and si-Grb2 539) were synthesized and transfected into BMDMs. The RT-PCR results indicated that the expression of Grb2 was significantly decreased after transfected by si-269 (Fig. 5F). The level of CD206, Arg-1, TGF-β1, and PDGF-AA mRNA were increased in the IL-4 group. Their expression in si-269 was significantly weaker compared to si-Control (Fig. 5G, H). Meanwhile, western blot result also indicated that the knockdown of Grb2 significantly reversed the M2 polarization induced by IL-4 (Fig. 5I). These results indicated that Grb2 was involved in the polarization of M2 macrophages.

### JCF5 inhibits macrophages M2 polarization by Grb2-mediated STAT6 phosphorylation

**Fig. 6:**
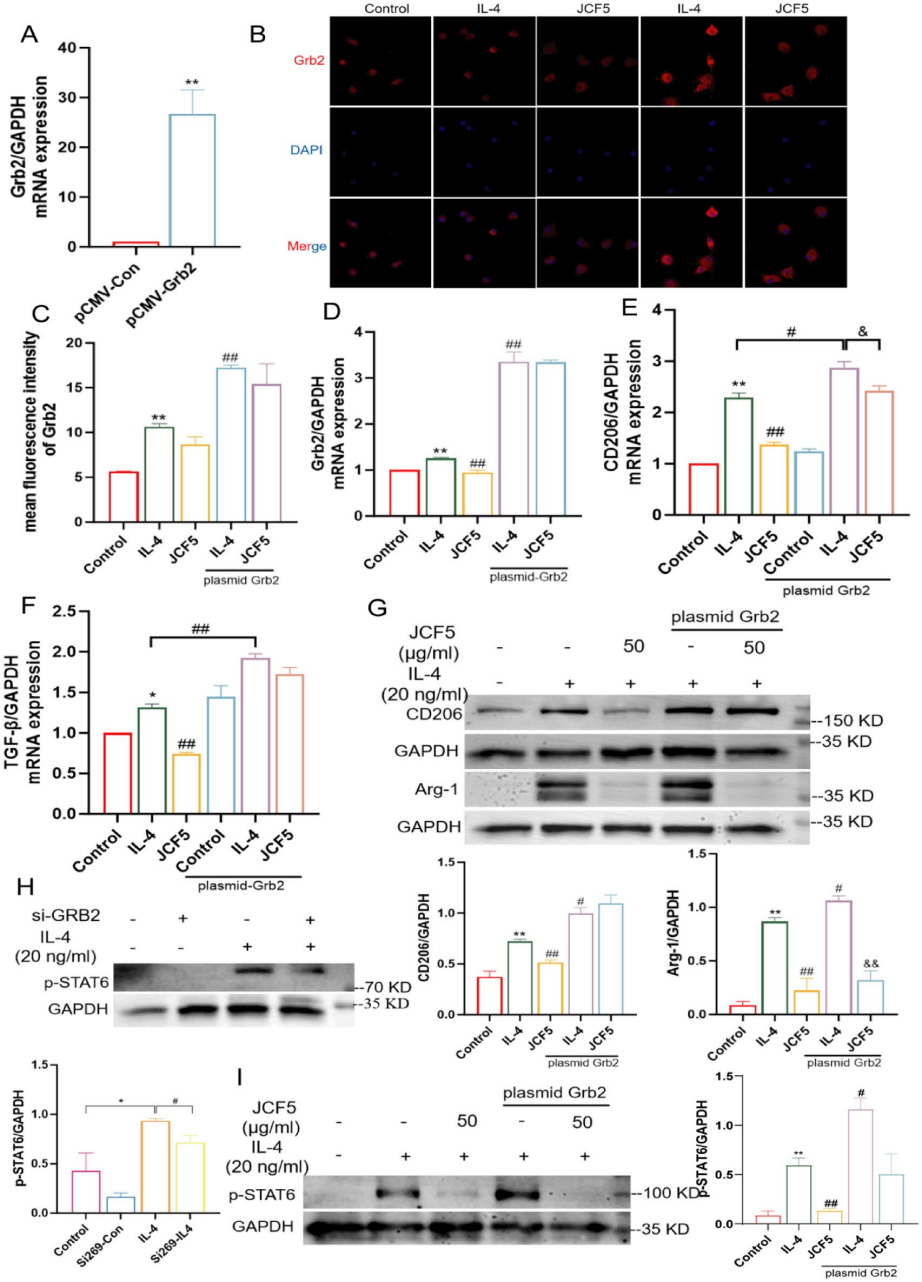
JCF5 inhibits macrophages M2 polarization by regulating STAT6 phosphorylation via Grb2. **A** The mRNA level of plasmid Grb2 in BMDMs cells. **B**, **C** The fluorescence intensity of Grb2 in BMDMs cells treated with plasmid Grb2 and JCF5. **D**–**F** The mRNA expression of Grb2, CD206 and TGF-β1 in BMDMs cells treated with plasmid Grb2 and JCF5. **G** The protein expression of Arg-1 and CD206 in BMDMs treated with plasmid Grb2 and JCF5. **H** The protein expression of p-STAT6 in BMDMs treated with si-Grb2 269. **I** The protein expression of p-STAT6 in BMDMs treated with plasmid Grb2 and JCF5. (*P < 0.05, **P < 0.01, vs. the Control; ^#^*P* < 0.05, ^##^*P* < 0.01, vs. the IL-4 group. ^&^*P* < 0.05, ^&&^*P* < 0.01, vs. the IL-4 in plasmid Grb2)

To reveal what role Grb2 plays in JCF5 inhibiting M2 macrophages, Grb2 overexpression plasmids were constructed and transfected into BMDMs. The RT-PCR result demonstrated that the Grb2 was overexpressed in BMDMs (Fig. 6A). The immunofluorescence showed that the expression of Grb2 were dramatically upregulated in plasmid-Grb2 group compared with plasmid-NC group (Fig. 6B-D). Meanwhile, the mRNA expressions of M2 marker, CD206 and TGF-β1, were significantly increased in IL-4 group of plasmid-Grb2 group compared with plasmid-NC group (Fig. 6E, F). And the protein level of Grb2, and Arg-1 were also notably increased. However, JCF5 significantly reversed these expression compared with IL-4 group (plasmid-Grb2) (Fig. 6G). These result indicated that the overexpression of Grb2 exhibited a significant capacity to counteract the effects of JCF5 on M2 macrophages. In addition, the expression of STAT6 phosphorylation was markedly down-regulated in BMDMs treated with si-Grb2 269 (Fig. 6H). Meanwhile, we found that the overexpression of Grb2 was significantly promoted the activation of STAT6, which revealed that JCF5 may exert an inhibitory effect on M2 macrophages polarization by downregulating the expression of Grb2 and regulating the activation of STAT6 (Fig. 6I).

### Hesperidin is the bioactive ingredient of JCF5 that targets Grb2

**Fig. 7:**
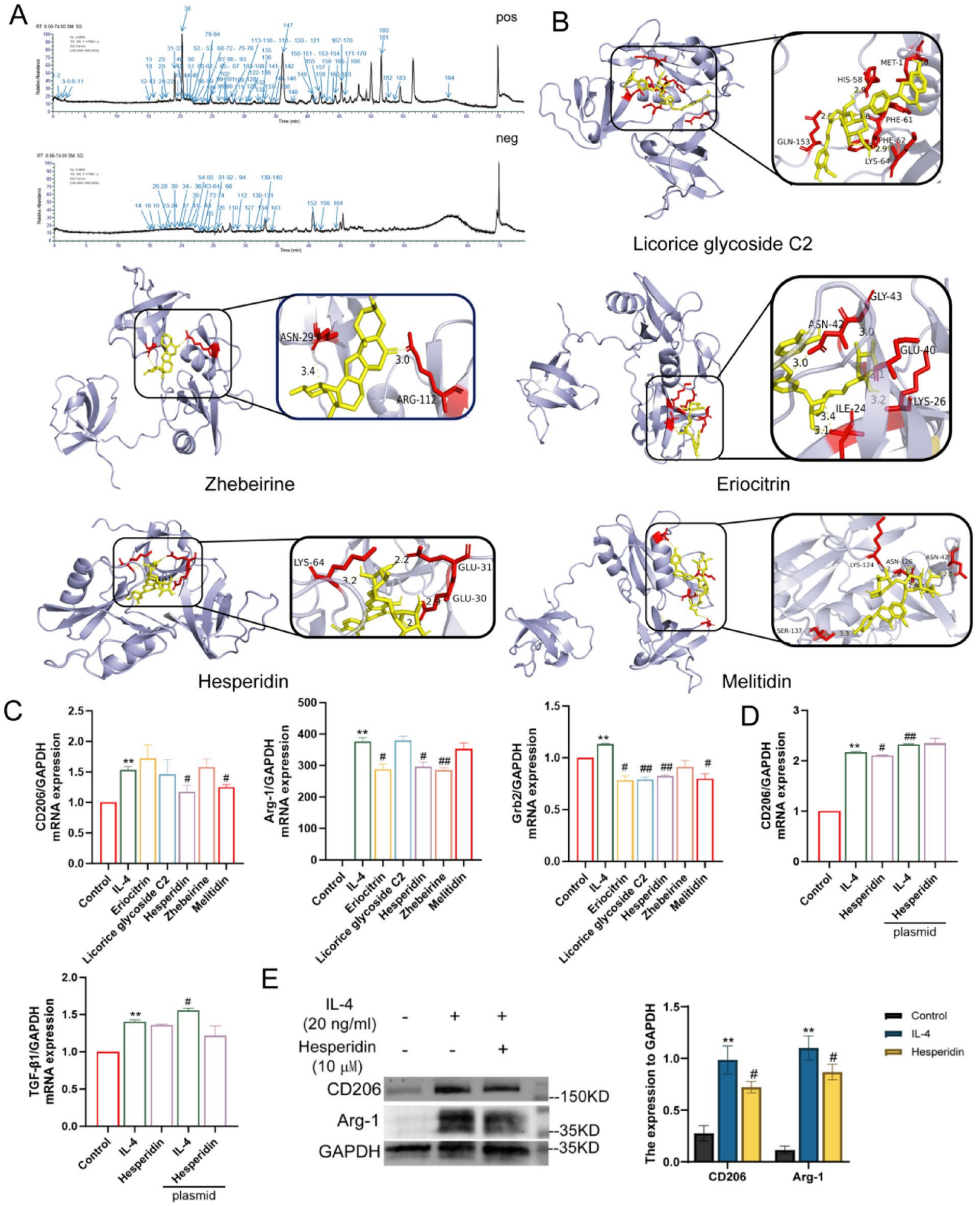
The effects of 5 active ingredients on inhibiting macrophage M2 polarization. **A** The total ion chromatogram of JCF5 in positive and negative modes. **B** The docking results of Grb2 with Licorice glycoside C2, Zhebeirine, Eriocitrin, Hesperidin and Melitidin. **C** The mRNA level of CD206, Arg-1 and Grb2 in BMDMs treated with 5 components. **D** The mRNA expression of CD206, TGF-β1 in BMDMs treated with plasmid Grb2 and Hesperidin. **E** The protein expression of CD206 and Arg-1 in BMDMs treated with Hesperidin. (*P < 0.05, **P < 0.01, vs. the Control; ^#^*P* < 0.05, ^##^*P* < 0.01, vs. the IL-4 group)

The chemical composition of JCF5 was determined by UPLC-Q/TOF–MS/MS, leading to the annotation of 186 small molecules (Fig. 7A and Table. S2). Molecular-docking simulations were subsequently employed to model the interaction landscape between these ligands and the Grb2 SH2/SH3 domains, yielding estimates of binding pose, affinity, and contact topology. Our results indicated that the top five small molecules—Licorice glycoside C2, Zhebeirine, Eriocitrin, Hesperidin and Melitidin—emerged as the top-ranking hits, each displaying favorable binding energies and extensive interface complementarity with Grb2 (Fig. 7B, C). Among them, Hesperidin significantly reduced mRNA levels of CD206, Arg-1, and Grb2. Meanwhile, the overexpression of Grb2 markedly attenuated the effect of Hesperidin on M2 macrophages (Fig. 7D).

## Discussion

Inflammation is the primary characteristic of the early stage in the lung tissue of silicosis model, which gradually progresses to tissue fibrosis and ultimately evolves into the fibrosis formation stage. Our preliminary research found that the Jinshui Chenfei formula (JCF) could lower the six-minute walking distance (6MWD), improve the exercise capacity, quality of life, and clinical symptoms of patients [15]. Meanwhile, we also found that inflammation is the main characteristic in the early stage in the lung tissue of silicosis rats, with gradually emerging tissue fibrosis progression, eventually evolving into the fibrosis formation stage based on our previous research on the evolution characteristics of silicosis rats. In this study, we established a silicosis rat model using disposable intratracheal instillation of SiO_2_ suspension, and observed the intervention effects of the Jinshui Chenfei formula on silicosis rats in three stages, respectively. And we found that JCF can significantly improve lung function, alleviate lung tissue pathology, reduce collagen deposition, and lower the marker levels of M2 macrophage polarization in different stages. At the same time, we used transcriptomics and drug target discovery techniques to discover that JCF may regulate STAT6 activation by binding to Grb2, thereby inhibiting M2 macrophage polarization. These findings uncovered the pharmacological mechanism and therapeutic potential of JCF in the treatment of silicosis fibrosis.

Macrophages, innate immune cells of the human body, are the critical effector cells in the development of silicosis, which are recruited and activated to respond to foreign excitants when SiO_2_ particles enter the lung tissue through respiration. It is reported that macrophages can polarize into M1 or M2 macrophages under different environments and stimuli [25]. M1 macrophages upregulate reactive oxygen intermediates and inducible nitric oxide synthase (iNOS), participating in helper T cell (Th) 1 immunity, killing and engulfing microorganisms to fight infections, and resisting pathogens. IL-4/IL-13-stimulated M2 macrophages up-regulate Arg-1 and mannose receptor (MR) on the surface of macrophages (CD206), while producing angiogenic mediators such as TGF-β and PDGF to promote angiogenesis, fibroblast recruitment and tissue repair while limiting inflammation [3]. In summary, the balance of M1/M2 macrophage polarization plays an important role in the development of silicosis, and M2 macrophages dominate the progression of silicosis fibrosis [26]. Therefore, this study evaluated the effect of JCF on M2 macrophage polarization in silicosis fibrosis through experiments in vitro and in vivo. These results showed that inflammatory cell infiltration was observed in the early stage, interalveolar thickening and blue collagen fibers appeared in the advanced lesions, and excessive collagen deposition was exposed during the fibrosis phase. However, JCF and tetrandrine treatment could significantly improve these pathologies and alleviate the deterioration of lung function indicators such as ventilation function at different stages. M2 macrophages release a large amount of pro-fibrotic mediators to promote tissue repair by activating fibroblasts to proliferate and differentiate into myofibroblasts. At the same time, they also secrete a large amount of collagen components such as Col-Ⅰ, causing excessive deposition of extracellular matrix and exacerbating fibrosis. Our JCF and tetrandrine reduced the levels of Col-Ⅰ and α-SMA in lung tissue and decreased collagen deposition. In addition, we evaluated the degree of M2 macrophage polarization by detecting the expression of Arg-1 and CD206, and our intervention could reduce their expression in rat lung tissue. These observations indicated that JCF may effectively alleviate collagen deposition and M2 macrophage polarization.

Combined with the adsorption properties of the macroporous resin, we obtained six JCF sub-fractions and screened them on IL-4-induced BMDMs. And it was found that JCF5 significantly reduced the levels of CD206 and Arg-1. Transcriptomic analyses revealed that JCF5 abrogated IL-4-induced M2 macrophage polarization, and orchestrated extracellular matrix remodeling, inflammatation, and redox homeostasis and JAK/STAT pathway. Importantly, STAT6 is a specific transcription factor for Th2 differentiation. Upon phosphorylation, STAT6 forms heterodimers that translocate to the nucleus, promote the release of profibrotic factors and thereby mediate inflammation and fibrosis [13]. Meanwhile, IL-4, IL-13 can also activate macrophages polarization, mediating inflammation and fibrosis. The STAT6 signal in CD11b positive cells promotes lung cancer progression by promoting IL-4 secretion and increasing M2 macrophages [5]. Inhibiting STAT6 phosphorylation can inhibit macrophages M2 polarization and delay the progression of renal fibrosis [9]. Therefore, STAT6 activation is a key signal driving macrophages towards M2 polarization, accelerating the development of tissue fibrosis. The results in our study found that JCF5 significantly inhibited M2 macrophages polarization and reduced the level of STAT6 phosphorylation.

DARTS LC–MS/MS profiling of JCF5-treated BMDMs identified its cellular binding proteome, within which STAT6 was conspicuously absent. We therefore constructed a protein–protein interaction network to probe whether any of the JCF5 targets engage STAT6 directly or indirectly. Our data uncovered direct or indirect interactions between STAT6 and Grb2, B2m, and Casp3. B2m, a nonglycosylated protein, is essential for MHC-I complex formation and peptide presentation [14]. There are few reports on its association with pulmonary fibrosis, and some studies have reported its application as an internal reference gene in macrophages [10]. Caspase-3 is a key executive factor in the apoptosis (programmed cell death) signal, playing a central role in regulating cell death, inflammatory responses, and various disease processes [20]. Its level were commonly used to evaluate the apoptosis of different types of cells in pulmonary fibrosis [21]. Grb2 is an adaptor protein, which composed by SH2 domain in the center and SH3 domain on each side [18]. In IL-4-induced M2 macrophage polarization, Grb2 acts as a critical amplifier. It is recruited to the activated IL-4 receptor via phosphorylated IRS2, initiating the Ras-MAPK (ERK) pathway. Nuclear ERK then phosphorylates STAT6 on a specific serine residue. This serine phosphorylation synergizes with JAK-mediated STAT6 tyrosine phosphorylation (Tyr641), thereby maximizing STAT6 transcriptional activity and the expression of M2-markers such as Arg1 and Mrc1. Thus, Grb2 bridges IL-4R to MAPK signaling, fine-tuning the master regulator STAT6 to form a potent axis driving M2 polarization [16]. Furthermore, previous studies have established that Gab proteins—Grb2-associated binding proteins belonging to the Dos/Gab subfamily of scaffold molecules—play essential roles in the regulation of cell growth, differentiation, and inflammation. Among them, Gab2 acts as a multifunctional signaling scaffold, frequently recruited by Grb2 to activated receptor complexes [7]. In the context of IL-4-induced M2 macrophages polarization, Gab2 contributes by positively modulating STAT6 activation. Notably, genetic ablation of Gab2 impairs STAT6 phosphorylation and ameliorates bleomycin-induced pulmonary fibrosis in mice [8].

Importantly, our study demonstrated that the expression of Grb2 in the gene and protein were significantly up-regulated in BMDMs induced by IL-4, and their level can be inhibited by JCF5. Meanwhile, the Grb2 gene silencing experiment was used to observe its role in driving macrophage M2 transformation, and our experiment confirmed that Grb2 is indeed involved in IL4 mediated M2 polarization in BMDMs. Next, the Grb2 overexpression promotes M2 polarization andblocks the effect of JCF5 and Hesperidin on IL-4-mediated BMDMs polarization.

## Conclusion

In summary, our study revealed the mechanism of JCF and Hesperidin, its representative components, inhibiting macrophage M2 polarization in the treatment of silicosis fibrosis by regulating the Grb2/STAT6 signaling, and confirmed the role of Grb2 in macrophage M2 polarization, which can serve as a potential therapeutic target for silicosis. However, this study did not investigate the regulatory effects of JCF on inflammation during the acute phase (0–7 days) in a SiO_2_-induced silicosis rat model, which is crucial for subsequent fibrosis development. The “formula (herb)-target-biomarker-effect-validation” five-dimensional integrated research model is an essential theoretical framework for elucidating the holistic concept of TCM formula [2]. However, our study did not perform in vivo genetic knockout experiments to further confirm the role of Grb2, which will be addressed in our future work.

## Supplementary Information

Supplementary material 1.

## Data Availability

No datasets were generated or analysed during the current study.

## Abbreviations

Arg-1: Arginase-1
CD206 (MRC1): Mannose receptor 1
Col-I: Collagen I
DARTS: Drug affinity responsive target stability
Grb2: Growth factor receptor-bound protein 2
JCF: Jinshui Chenfei formula
PDGF-AA: Platelet-derived growth factor AA
STAT6: Signal transducer and activator of transcription 6
TGF-β: Transforming growth factor-β
