# Supplementary material for: Jinshui Chenfei formula alleviates SiO2-induced pulmonary fibrosis by inhibiting macrophage M2 polarization via Grb2/STAT6 in rats
Source: Chin Med. 2026 Mar 16;21:85. doi: 10.1186/s13020-026-01339-7 (PMC12990592; doi:10.1186/s13020-026-01339-7)
Supplement: Supplementary file 1 — Supplementary material 1. [file 13020_2026_1339_MOESM1_ESM.docx]

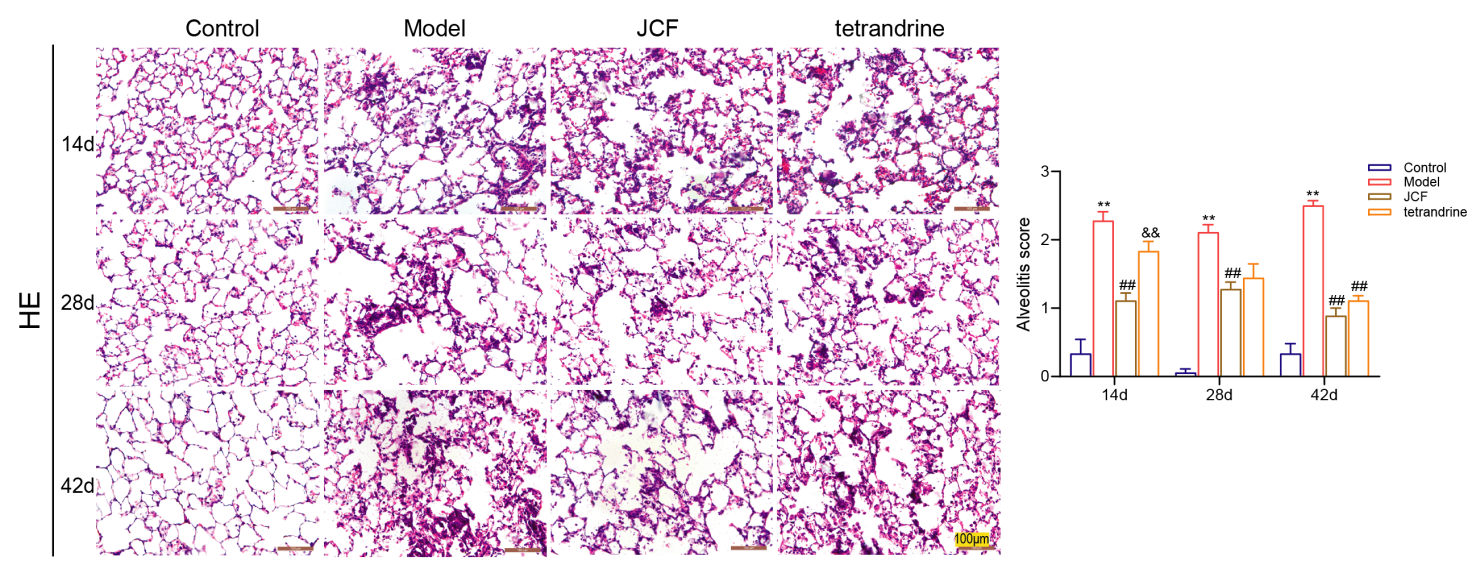


**Fig. S1 The lung tissue pathology of JCF treating silicosis rats.**

(**P* < 0.05, ***P* < 0.01, vs. the Control; ^#^*P* < 0.05, ^##^*P* < 0.01, vs. the Model; ^&^*P* < 0.05, ^&&^*P* < 0.01, vs. the JCF group)


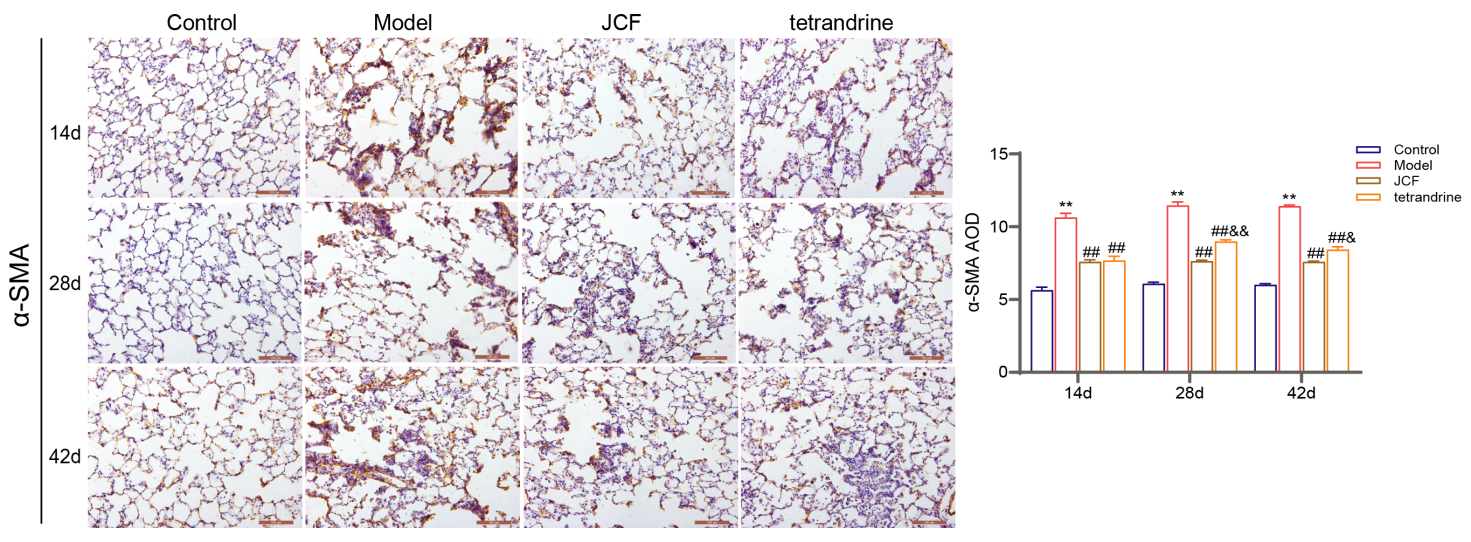


**Fig. S2 The expression of α-SMA in lung tissue of silicosis rats.**

(**P* < 0.05, ***P* < 0.01, vs. the Control; ^#^*P* < 0.05, ^##^*P* < 0.01, vs. the Model; ^&^*P* < 0.05, ^&&^*P* < 0.01, vs. the JCF group)

**Fig. S3 The safe concentration of JCF5 in MH-S and RAW264.7.**

**
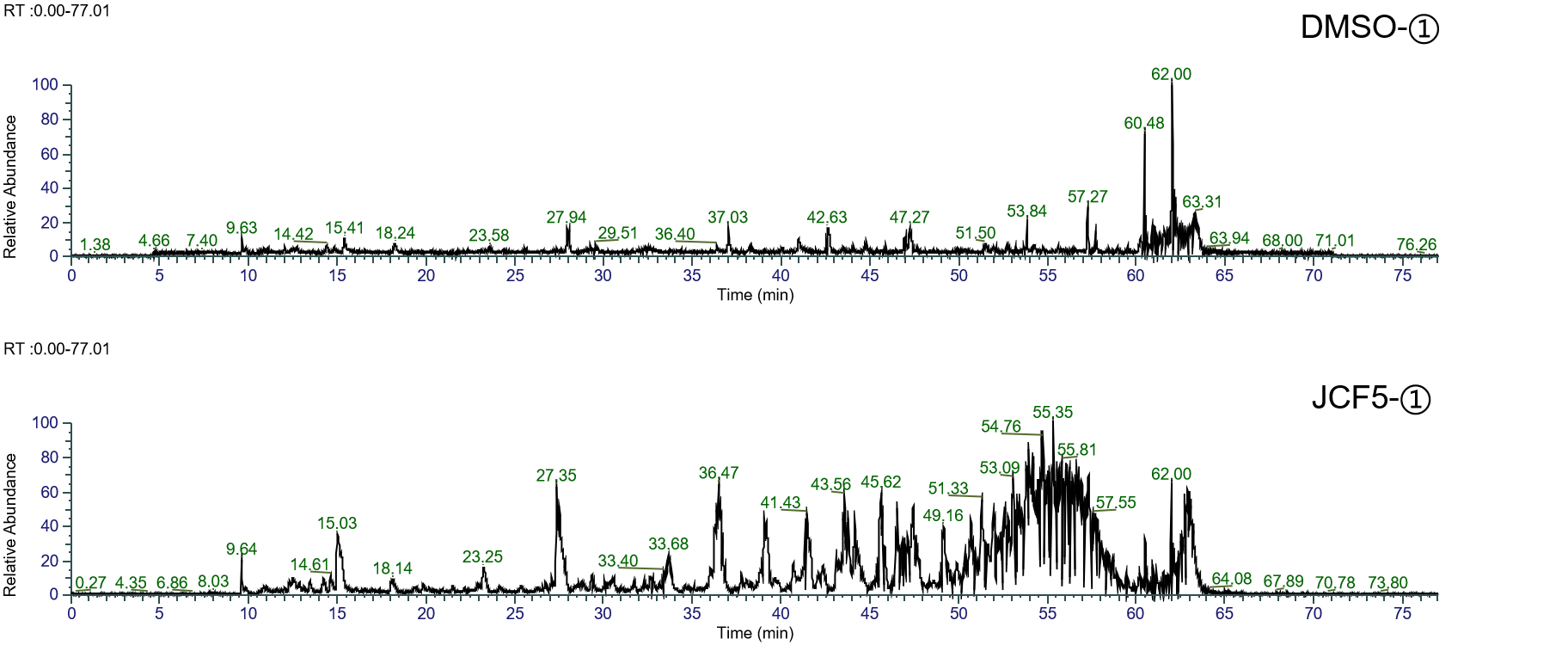
**

**
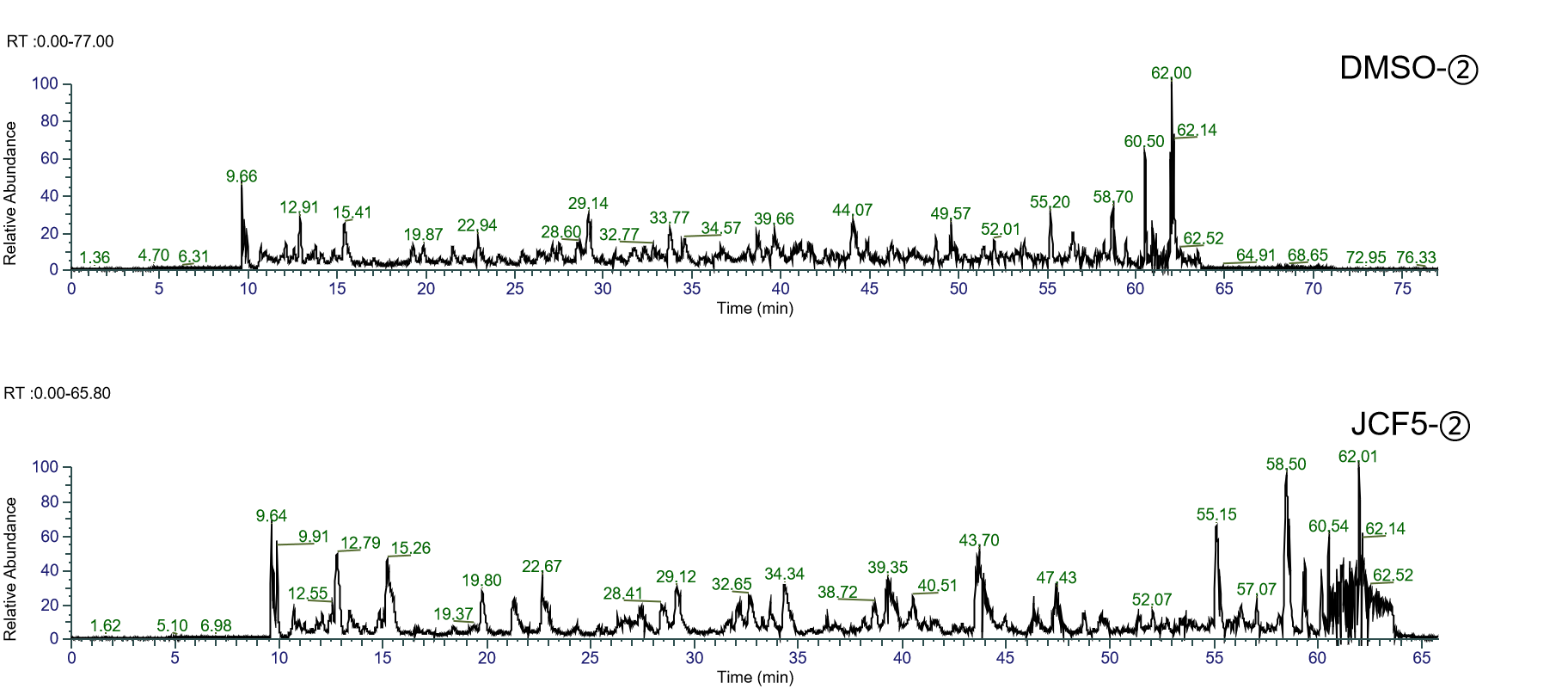
**

**
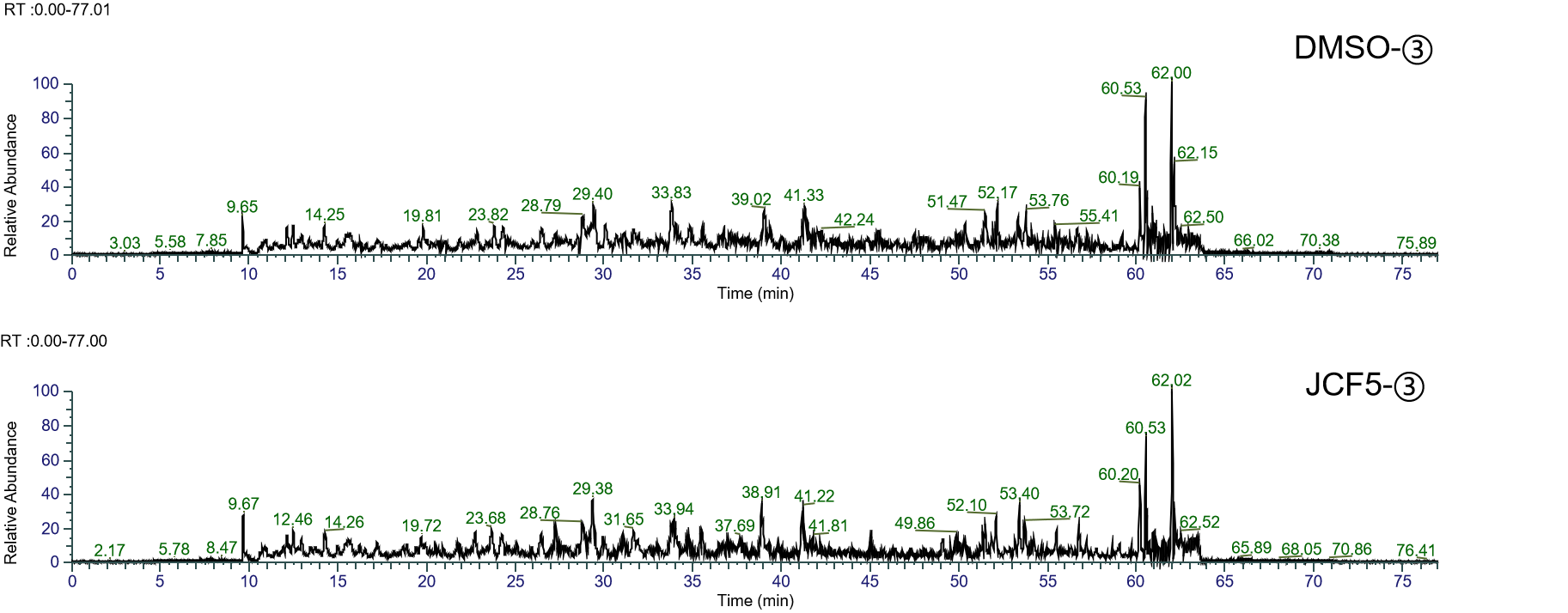
**

**
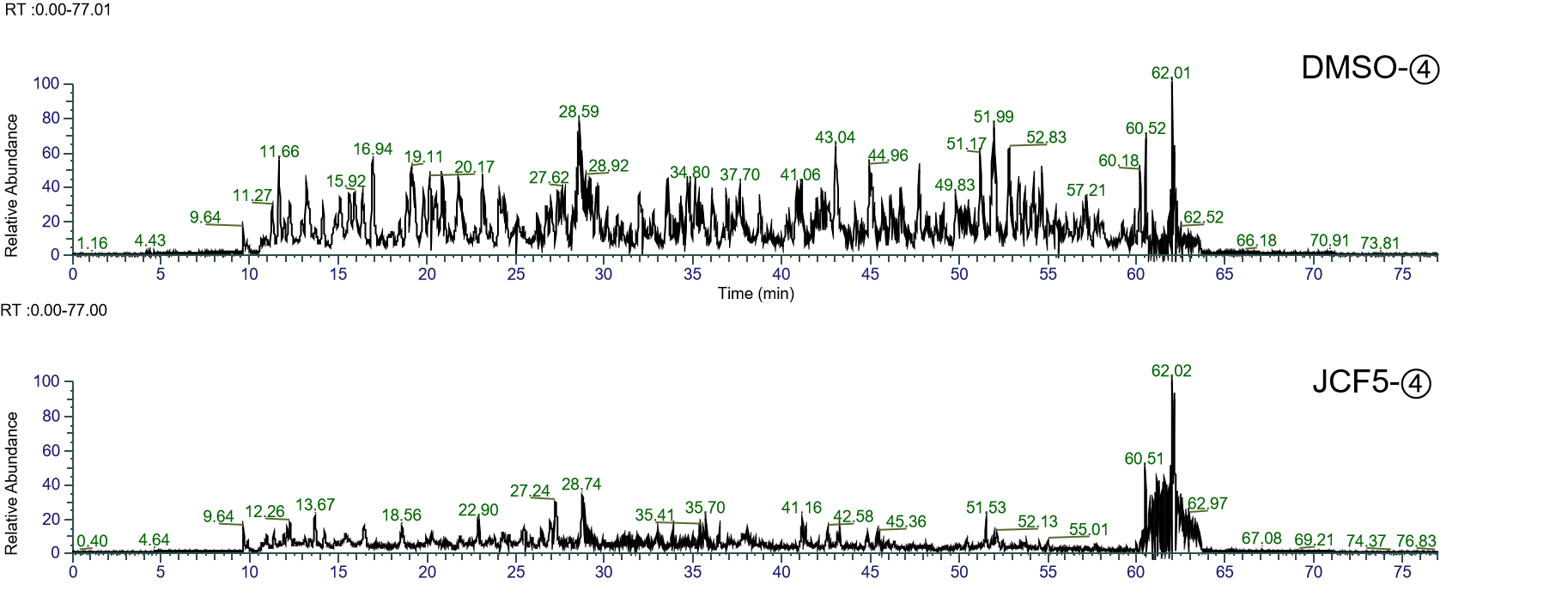
**

**Fig. S4 The total ion current chromatograms of the four gel slices.(positive ion mode)**

**Table S1 The target proteins information obtained from DARTS-LC-MS/MS.**

| **Gel** | **Protein** | **Coverage％** | **Molecular mass**  **(kDa)** | **LFQ intensity of JCF5/Con** |
| --- | --- | --- | --- | --- |
| ① | Dbi | 81.6 | 10.00 | 1.521544728 |
|  | Banf1 | 59.6 | 10.10 | 3.295521662 |
|  | Sf3b5 | 38.4 | 10.12 | 1.795207748 |
|  | Rpl37a | 42.4 | 10.28 | 1.862148311 |
|  | S100a8 | 49.4 | 10.29 | 1.705814966 |
|  | Uqcrh | 39.3 | 10.44 | 3.218552354 |
|  | Timm13 | 53.7 | 10.46 | 2.836319168 |
|  | Mrps21 | 31 | 10.56 | 1.687150049 |
|  | Dynlrb1 | 38.5 | 10.99 | 1.564630091 |
|  | Mrps36 | 36.3 | 11.10 | 1.960319400 |
|  | Vma21 | 34.7 | 11.37 | 2.234543987 |
|  | Hist1h4a | 68.9 | 11.37 | 3.085646823 |
|  | Txn | 66.7 | 11.68 | 1.643839025 |
|  | Mrpl57 | 21.6 | 11.95 | 1.651429521 |
|  | Atpif1 | 43.4 | 12.16 | 1.727511299 |
|  | Rpl36 | 21.9 | 12.25 | 2.309911635 |
|  | Phf5a | 64.5 | 12.41 | 1.843355472 |
|  | Rpl35a | 48.2 | 12.55 | 1.573472042 |
|  | Rpl30 | 58.3 | 12.78 | 1.824055066 |
|  | Rps26 | 27 | 13.02 | 1.694093230 |
|  | Atp6v1f | 42.9 | 13.37 | 1.705333684 |
|  | H2afv;H2afz | 31.2 | 13.51 | 4.005490700 |
|  | B2m | 50.4 | 13.78 | 1.762475265 |
|  | Hist1h2bc;Hist2h2bb;Hist1h2bh;Hist1h2bm | 58.7 | 13.91 | 2.932968659 |
|  | Hist2h2ac;Hist2h2aa1 | 58.1 | 13.99 | 6.469900422 |
|  | Hist1h2ah;H2afj;Hist1h2ak;Hist1h2af;Hist3h2a;H2afx | 57.7 | 14.14 | 3.470280552 |
|  | Trmt112 | 16 | 14.14 | 1.946493312 |
|  | Nhp2l1 | 40.6 | 14.17 | 1.742384073 |
|  | Mmgt1 | 8.4 | 14.68 | 1.650927442 |
|  | Lgals1 | 95.6 | 14.87 | 1.510275599 |
|  | Hist1h3b;Hist1h3a;H3f3a;H3f3c | 32.4 | 15.39 | 3.683019345 |
| ② | H2afx | 67.8 | 15.142 | 2.370984192 |
|  | Mrps16 | 28.9 | 15.192 | 1.527355163 |
|  | Hist1h3b | 68.4 | 15.388 | 3.340109049 |
|  | Rps24 | 29.3 | 15.423 | 2.078300151 |
|  | Rpl28 | 52.6 | 15.733 | 1.939832445 |
|  | Atp6v0c | 34.2 | 15.808 | 1.541943554 |
|  | Rpl32 | 55.6 | 15.86 | 2.322586311 |
|  | Rpl27a | 31.1 | 16.605 | 2.347497597 |
|  | Ostc | 14.1 | 16.815 | 1.645016462 |
|  | Calml3 | 46.3 | 16.837 | 2.179154202 |
|  | Myl6 | 60.3 | 16.93 | 2.642788559 |
| ③ | Rps3 | 83.5 | 26.674 | 1.620367064 |
|  | Mrto4 | 51 | 27.545 | 1.771430403 |
|  | Srsf1 | 58.9 | 27.744 | 2.803045575 |
|  | Rpl8 | 33.1 | 28.024 | 2.475926056 |
|  | Snrpa1 | 19.2 | 28.357 | 2.017935702 |
|  | Atp6v1d | 54.3 | 28.369 | 1.647888718 |
|  | Rps4x | 60.5 | 29.597 | 2.30819509 |
|  | Rpl7a | 32.7 | 29.976 | 1.797830436 |
|  | Cops7a | 30.5 | 30.224 | 1.52842727 |
|  | Nit2 | 38 | 30.501 | 1.742726111 |
|  | Vdac3 | 51.2 | 30.752 | 1.593127921 |
|  | Srsf7 | 30.7 | 30.817 | 1.508886081 |
|  | Anp32b | 29.4 | 31.078 | 3.918846223 |
|  | Rpl7 | 62.6 | 31.419 | 1.97835882 |
|  | Abhd17b | 26.4 | 32.2 | 1.532125206 |
|  | Tra2a | 15.3 | 32.316 | 2.013574329 |
|  | Cdk5 | 10.6 | 33.288 | 1.624202544 |
|  | Set | 9.3 | 33.377 | 1.661179968 |
|  | Mgll | 44.6 | 33.387 | 1.59908442 |
|  | Auh | 16.2 | 33.395 | 2.744256456 |
|  | Mtch2 | 26.7 | 33.499 | 1.557588652 |
|  | Abhd11 | 10.4 | 33.56 | 1.693123891 |
|  | Tra2b | 28.5 | 33.665 | 3.118588213 |
|  | Eif2b1 | 8.9 | 33.816 | 1.822074096 |
|  | Slc25a11 | 58 | 34.155 | 1.767242012 |
|  | Rplp0 | 26.5 | 34.216 | 2.147093519 |
|  | Fbl | 42.2 | 34.306 | 1.890318071 |
|  | Emc2 | 32 | 34.934 | 1.567101667 |
|  | Cyc1 | 16 | 35.327 | 1.583344287 |
| ④ | Grb2 | 22.6 | 25.238 | 2.254269898 |
|  | Atp6v1e1 | 66.4 | 26.157 | 4.684949623 |
|  | Psma5 | 18.3 | 26.411 | 1.86192618 |
|  | Clic1 | 61.4 | 27.013 | 1.90028083 |
|  | Psme2 | 47.3 | 27.057 | 1.972362642 |
|  | Clec7a | 16 | 27.42 | 1.687740325 |
|  | Lgals3 | 43.9 | 27.515 | 1.787476147 |
|  | Rpl8 | 30.4 | 28.024 | 2.747990788 |
|  | Ywhah | 45.5 | 28.211 | 1.544620952 |
|  | Ywhag | 46.2 | 28.302 | 1.500617452 |
|  | Atp6v1d | 57.5 | 28.369 | 2.061826308 |
|  | Exosc6 | 21.6 | 28.37 | 2.870064482 |
|  | Ado | 9.8 | 28.372 | 2.010157804 |
|  | Fcgr4 | 14.9 | 28.398 | 1.561645135 |
|  | Tpm4 | 44.4 | 28.467 | 2.854683857 |
|  | Pcna | 73.2 | 28.785 | 1.805704425 |
|  | Ywhae | 65.9 | 29.174 | 2.078828829 |
|  | Psma4 | 11.9 | 29.47 | 1.699369032 |
|  | Psma1 | 66.2 | 29.546 | 2.878821684 |
|  | Rps3a | 67.4 | 29.885 | 2.068635834 |
|  | Rpl7a | 32.3 | 29.976 | 1.966279218 |
|  | Lasp1;Nebl | 6.5 | 29.994 | 1.722374577 |
|  | Mrpl28 | 10.9 | 30.169 | 4.938722656 |
|  | Cbr1 | 59.6 | 30.641 | 2.115909091 |
|  | Srsf7 | 30.7 | 30.817 | 5.257433291 |
|  | Hnrnpab | 42.5 | 30.831 | 1.510011507 |
|  | Srsf5 | 19.7 | 30.891 | 3.887578785 |
|  | M6pr | 18.3 | 31.172 | 1.709729555 |
|  | Hvcn1 | 30.1 | 31.242 | 2.398012552 |
|  | Eef1d | 48.4 | 31.293 | 1.947061485 |
|  | Esd | 74.1 | 31.319 | 1.810105705 |
|  | Capzb | 57.8 | 31.345 | 1.550326077 |
|  | Rpl7 | 55.6 | 31.419 | 4.513839603 |
|  | Casp3 | 6.9 | 31.474 | 1.740041929 |
|  | Pitpnb | 35.4 | 31.487 | 1.633861461 |
|  | Vdac2 | 56.6 | 31.732 | 2.090836501 |
|  | Mlec | 39.5 | 32.342 | 2.213240682 |
|  | Vdac1 | 61.1 | 32.351 | 2.130989476 |
|  | Ergic1 | 29.7 | 32.562 | 2.687515763 |
|  | Ppa1 | 36 | 32.667 | 1.711072539 |
|  | Lactb2 | 18.8 | 32.754 | 2.009027728 |
|  | Hsd17b11 | 19.8 | 32.88 | 1.813736365 |
|  | Atp5c1 | 27.5 | 32.886 | 2.664108382 |
|  | Golph3l | 12.3 | 32.905 | 1.60254037 |
|  | Tsnax | 23.1 | 32.926 | 2.132287539 |
|  | Tpm3 | 53 | 32.994 | 4.076909988 |
|  | Stx2 | 8.3 | 33.177 | 1.972153804 |
|  | Napa | 74.2 | 33.189 | 1.695712183 |
|  | Bpnt1 | 38.3 | 33.196 | 1.685683912 |
|  | Phb2 | 68.2 | 33.296 | 2.702577484 |
|  | Glod4 | 41.9 | 33.316 | 1.600925037 |
|  | Mtch2 | 12.5 | 33.499 | 1.741085271 |
|  | Rpl6 | 43.6 | 33.509 | 2.02126407 |
|  | Blvra | 70.2 | 33.524 | 1.663241631 |
|  | Mrpl15 | 30.5 | 33.541 | 3.631470272 |
|  | Ppcs | 9.6 | 33.794 | 1.910915012 |
|  | Tmx2 | 7.5 | 33.942 | 2.391739895 |
|  | Srm | 28.8 | 33.995 | 1.922776985 |
|  | Ctsz | 45.1 | 33.996 | 2.240233036 |
|  | Dhrs1 | 31.3 | 34.005 | 1.825827815 |
|  | Cyb5r3 | 54.2 | 34.127 | 1.723412384 |
|  | Stx4 | 27.5 | 34.165 | 1.878154327 |
|  | Hnrnpa1 | 21.6 | 34.196 | 1.683227008 |
|  | Hmgcl | 9.8 | 34.238 | 2.276419446 |
|  | Tomm34 | 25.2 | 34.278 | 2.552028688 |
|  | Arpc2 | 66.7 | 34.357 | 3.988512637 |
|  | Rpl5 | 49.8 | 34.4 | 3.029311564 |
|  | Akr1e2 | 26.2 | 34.46 | 2.490577266 |
|  | Hadh | 36 | 34.463 | 1.802145787 |
|  | Psmd14 | 40 | 34.577 | 1.864144264 |
|  | Stap1 | 24.2 | 34.627 | 1.591160221 |
|  | Hsd17b12 | 45.2 | 34.741 | 1.955335318 |
|  | Prps2 | 31.4 | 34.786 | 1.751999158 |
|  | Prps1 | 27.7 | 34.834 | 2.549865451 |
|  | Lrrc59 | 23.1 | 34.877 | 1.540308652 |
|  | Pura | 8.7 | 34.883 | 1.578188687 |
|  | Stub1 | 21.7 | 34.909 | 1.748457062 |
|  | Emc2 | 15.5 | 34.934 | 3.893316399 |
|  | Spg21 | 28.9 | 34.952 | 1.718133003 |
|  | Etfa | 28.2 | 35.009 | 1.91206197 |

**Table S2 Chemical composition in JCF5 detected by using UHPLC-Q-Orbitrap MS**

| **No.** | **Compounds** | **RT** | **Formula** | **Detected** | **Error (ppm)** | **MS/MS** | **Polarity** |
| --- | --- | --- | --- | --- | --- | --- | --- |
|  | Glutamic acid | 1.33 | C5H9NO4 | 148.06 | 4.6 | 84.0443; 56.0498 | pos |
|  | Benzoic acid | 1.37 | C7H6O2 | 123.04 | -2.37 | 105.0436; 95.0490 | pos |
|  | Dianthoside | 1.39 | C12H16O8 | 289.09 | 5.4 | 127.0388 | pos |
|  | M-Hydroxybenzoic Acid or its isomer (a) | 1.46 | C7H6O3 | 139.04 | 5.39 | 121.0282; 95.0490; 65.0386 | pos |
|  | Quinic acid | 1.48 | C7H12O6 | 193.07 | 4.54 | 175.0597; 157.0494; 147.0650 | pos |
|  | Malic Acid | 1.64 | C4H6O5 | 135.03 | -6.32 | 85.0840; 79.0541; 71.0127; 57.0338; 55.0182; 53.0225 | pos |
|  | Vitamin C | 1.66 | C6H8O6 | 177.04 | 4.32 | 149.0596; 121.0647; 103.0538; 91.0540 | pos |
|  | M-Hydroxybenzoic Acid or its isomer (b) | 1.71 | C7H6O3 | 139.04 | 5.39 | 121.0282; 95.0490; 65.0386 | pos |
|  | Synephrine | 1.79 | C9H13NO2 | 168.1 | 4.88 | 150.0912; 135.0677; 119.0490; 107.0490 | pos |
|  | Citric Acid/Isocitric acid (a) | 1.8 | C₆H₈O₇ | 191.02 | 9.7 | 111.0071; 87.0070; 85.0277; 57.0328 | pos |
|  |  | 1.81 | C₆H₈O₇ | 193.03 | 6.72 | 157.0130; 139.0025; 129.0189; | pos |
|  | Adenine | 1.83 | C5H5N5 | 136.06 | 5.58 | 136.0618; 119.0350; 109.0507; 94.0399; 92.0243 | pos |
|  | Aposcopolamine or its isomer (a) | 15.14 | C17H19NO3 | 286.14 | 9.47 | 131.049 | pos |
|  | Aposcopolamine or its isomer (b) | 15.35 | C17H19NO3 | 286.14 | 9.47 | 131.0491 | pos |
|  | 5-p-Coumaroylquinic acid or its isomer (b) | 15.45 | C16H18O8 | 337.09 | 8.14 | 191.0552; 163.0388 | neg |
|  | Methyl Phenylacetate | 15.85 | C9H10O2 | 151.08 | 6.88 | 92.0575; 123.0804; 105.0699; 95.0855 | pos |
|  | 5-p-Coumaroylquinic acid or its isomer (c) | 15.86 | C16H18O8 | 337.09 | 8.14 | 191.0551; 171.0283; 163.0387 | neg |
|  | 6-O-galloylsalidroside or its isomer (b) | 15.86 | C21H24O11 | 451.12 | 7.42 | 313.0568; 271.0457; 169.0128; 125.0228 | pos |
|  | 6-(3-Hydroxy-3-methylglutaroyl)-vicenin-2 or its isomer (a) | 16.12 | C33H38O19 | 737.19 | -9.92 | 473.1075; 353.0663 | pos |
|  | Rhodiosin/Quercetin-3-O-rhamnosyl (1→2)-rhamnoside or their isomer (a) | 16.88 | C27H30O16 | 609.14 | 5 | 301.0333; 255.0306; 151.0019 | neg |
|  | Eriocitrin | 16.98 | C27H32O15 | 595.17 | 4.69 | 287.0589; 193.0114 | pos |
|  | Cassythicine | 17.09 | C19H19NO4 | 326.14 | 5.14 | 311.1151 | pos |
|  | Fenfangjine G | 17.09 | C22H27NO8 | 434.18 | 4.86 | 374.1604; 314.1387 | pos |
|  | Kaempferol-3-O-rhamnosyl (4,1)-rhamnoside/Kaempferitrin/Kaempferol-3-O-rhamnosyl (2,1)-rhamnoside | 17.15 | C27H30O14 | 577.15 | 6.64 | 431.1016; 285.0401; 283.0251; 255.0292 | neg |
|  | Vitexin | 17.17 | C21H20O10 | 431.1 | 6.01 | 269.0451 | neg |
|  | Rosmarinic acid-3-O-glucoside(a) | 17.61 | C24H26O13 | 521.13 | 0.86 | 359.0766; 323.0771; 197.0447; 161.0231 | pos |
|  | Liguiritigenin-7-O-apiosyl-4’-O-glucoside/isoliquiritinapioside or their isomer (a) | 17.61 | C26H30O13 | 549.16 | -2.87 | 255.0661; 153.0180; 135.0073; 119.0487 | neg |
|  | Liguiritigenin-7-O-apiosyl-4’-O-glucoside/Isoliquiritinapioside or their isomer (b) | 17.8 | C26H30O13 | 549.16 | -2.87 | 255.0660; 153.0180; 135.0073; 119.0487 | neg |
|  | Liquiritin/Neoliquiritin/Neoisoliquiritin (a) | 17.85 | C21H22O9 | 417.12 | 3.56 | 255.0660; 135.0073 | neg |
|  | Liquiritigenin/Isoliquiritigenin or their isomer (a) | 17.86 | C15H12O4 | 257.08 | 2.96 | 147.0439; 137.0233 | pos |
|  | Neodiosmin | 18.93 | C28H32O15 | 607.17 | 0.68 | 299.0567 | neg |
|  | Verticinone-3-glucoside or its isomer (b) | 18.97 | C33H53NO8 | 592.38 | 1.9 | 594.3730; 412.3197 | pos |
|  | Hesperidin | 19.26 | C28H34O15 | 611.2 | 3.67 | 303.3867; 195.0289; 153.0188 | pos |
|  | Peimisine or its isomer (a) | 19.43 | C27H41NO3 | 428.32 | 1.9 | 412.3204 | pos |
|  | Hesperetin or its isomer (b) | 19.48 | C16H14O6 | 301.07 | -3.65 | 301.0719; 300.9989 | neg |
|  | Rosin | 19.48 | C15H20O6 | 297.13 | 0.11 | 135.0798 | pos |
|  | Rhodioloside B or its isomer (b) | 19.5 | C22H38O12 | 493.23 | 1.64 | 131.0337; 113.0232 | neg |
|  | Prunin/5-Hydroxyliquiritin | 19.7 | C21H22O10 | 433.11 | 0.48 | 271.0611 | neg |
|  | Rosmarinic acid or its isomer (b) | 20.18 | C18H16O8 | 359.08 | -2.3 | 161.0226; 133.0277 | pos |
|  | Rhodiooctanoside | 20.48 | C19H36O10 | 423.22 | -1.15 | 291.1809; 233.0664; 161.0436; 131.0331 | neg |
|  | Isoverticine/Zhebeinine (a) | 20.51 | C27H45NO3 | 432.35 | 0.25 | 414.3359 | pos |
|  | Hesperetin or its isomer (c) | 20.66 | C16H14O6 | 301.07 | 5.78 | 164.0097; 151.0019; 136.0151 | neg |
|  | Liguiritigenin-7-O-apiosyl-4’-O-glucoside/isoliquiritinapioside or their isomer (d) | 20.69 | C26H30O13 | 551.18 | -0.03 | 257.0801 | pos |
|  | Rhodioloside B or its isomer (c) | 20.72 | C22H38O12 | 493.23 | -4.35 | 447.2227; 191.0545 | neg |
|  | Apigenin or its isomer (b) | 20.85 | C15H10O5 | 271.06 | 1.52 | 153.0179 | pos |
|  | Peimisine or its isomer (b) | 20.89 | C27H41NO3 | 428.32 | 1 | 410.3046 | pos |
|  | Luteolin or its isomer (a) | 20.93 | C15H10O6 | 287.05 | -0.29 | 241.0485; 153.0179 | pos |
|  | Kaempferol-7-O-rhamnoside/Luteolin-7-O-rhamnoside/Kaempferol-3-O-rhamnoside (a) | 20.94 | C21H20O10 | 431.1 | -1.66 | 285.039; 255.0289; 227.0388 | pos |
|  | Rhodiosin/Quercetin-3-O-rhamnosyl (1→2)-rhamnoside or their isomer (c) | 21.1 | C27H30O16 | 609.15 | -2.41 | 301.0346 | pos |
|  | Daidzein or is isomer (a) | 21.34 |  | 255.07 | -8.37 |  | pos |
|  | Quercetin-3-O-glucuronide | 21.42 | C21H18O13 | 477.07 | -3.63 | 301.0345; 267.0655 | pos |
|  | Isoliquiritin | 21.5 | C21H22O9 | 417.12 | 0.93 | 255.0660; 135.0073 | pos |
|  | Hexandraside F/Maohuoside B/sagittasine A | 21.63 | C39H50O20 | 839.3 | 0.74 | 369.1322; 313.0696 | pos |
|  | Liquiritin/Neoliquiritin/Neoisoliquiritin (b) | 21.79 | C21H22O9 | 419.13 | 0.8 | 257.0809; 239.0691 | pos |
|  | Benzoyl-Oxypaeoniflorin/Mudanpioside C or their isomer (b) | 22 | C30H32O13 | 599.18 | -2.18 | 281.0657; 137.0226; 121.0277 | neg |
|  | Liquiritigenin/Isoliquiritigenin or their isomer (b) | 22.01 | C15H12O4 | 255.07 | 1.07 | 119.0492 | neg |
|  | 5,7-Dimethoxycoumarin (a) | 22.01 | C11H10O4 | 207.07 | -0.51 | 192.0411; 149.0229; 121.0281 | pos |
|  | Baohuoside VI or its isomer (a) | 22.05 | C39H50O19 | 823.3 | -0.18 | 369.1322; 313.0696 | pos |
|  | Peiminine | 22.09 | C27H43NO3 | 430.33 | 0.21 | 412.3198 | pos |
|  | Epimedin A | 22.15 | C39H50O20 | 839.3 | 0.74 | 369.1322; 313.0696 | pos |
|  | Epimedin B | 22.56 | C38H48O19 | 809.29 | 0.41 | 369.1322; 313.0696 | pos |
|  | Icaritin/Gancaonin B or their isomer (a) | 22.58 | C21H20O6 | 369.13 | 0.78 | 313.0698 | pos |
|  | Icariside I/Wushan icariin or their isomer (a) | 22.58 | C27H30O11 | 531.19 | 1.43 | 369.1323; 313.0697 | pos |
|  | Benzoyl-Oxypaeoniflorin/Mudanpioside C or their isomer (c) | 22.75 | C30H32O13 | 599.18 | -1.88 | 281.0659; 137.0226; 121.0277 | neg |
|  | Poncirin/Didymin or their isomer (b) | 22.8 | C28H34O14 | 593.19 | 1.71 | 287.0572 | neg |
|  | Baohuoside VI or its isomer (b) | 22.9 | C39H50O19 | 823.3 | 0.49 | 369.1322; 313.0696 | pos |
|  | 2″-O-rhamnosylicariside II or its isomer (a) | 22.91 | C33H40O14 | 659.24 | 2.33 | 351.0863; 323.0908 | neg |
|  | Daidzein or is isomer (b) | 22.99 | C15H10O4 | 253.05 | -3.23 | 227.0697; 199.0752; 137.023 | pos |
|  | 20(R)-Ginsenoside Rg2 | 23 | C42H72O13 | 785.5 | 0.42 | 441.3776; 423.3607; 405.3488 | pos |
|  | Longispinogenin or its isomer (a) | 23 | C30H50O3 | 459.38 | 0.38 | 441.3706; 423.3629 | pos |
|  | Sagittasine C or its isomer (c) | 23.02 | C33H40O16 | 693.24 | 2.14 | 385.1273; 329.0648 | pos |
|  | Daidzein or is isomer (b) | 23.05 | C15H10O4 | 255.07 | 0.79 | 227.0697; 199.0752; 137.023 | pos |
|  | 20(R)-Ginsenoside Rh1 or its isomer (a) | 23.09 | C36H62O9 | 639.45 | 0.93 | 441.3704; 423.3606; 305.3515 | pos |
|  | Licorice glycoside A/Licorice glycoside C1/Licorice glycoside C2(a) | 23.16 | C36H38O16 | 725.21 | 7.51 | 549.1593; 531.1458; 255.0642 | neg |
|  | Mudanpioside H | 23.17 | C30H32O14 | 617.18 | -0.27 | 267.0585; 105.0335 | neg |
|  | Poncirin/Didymin or their isomer (c) | 23.17 | C28H34O14 | 595.2 | 0.59 | 449.1430; 287.0903; 195.0284 | pos |
|  |  | 23.18 | C28H34O14 | 593.19 | -2.68 | 285.076 | pos |
|  | Licorice glycoside A/Licorice glycoside C1/Licorice glycoside C2 (a) | 23.19 | C36H38O16 | 727.22 | -2.27 | 257.08 | pos |
|  | Licochalcone B or its isomer (b) | 23.21 | C16H14O5 | 287.09 | 0.22 | 245.0816; 193.0483; 121.0281 | pos |
|  | Isoverticine/Zhebeinine (b) | 23.49 | C27H45NO3 | 432.35 | 0.88 | 414.3356 | pos |
|  | Sagittatoside A/Icariin (b) | 23.8 | C33H40O15 | 677.24 | -0.22 | 531.1851; 369.1324; 313.0699 | pos |
|  | Icaritin/Gancaonin B or their isomer (b) | 23.82 | C21H20O6 | 369.13 | 0.61 | 313.0697 | pos |
|  | Icariside I/wushan icariin or their isomer (b) | 23.82 | C27H30O11 | 531.19 | 0.51 | 369.1324; 313.0698 | pos |
|  | Sudachiin C or Sudachiin B (b) | 23.88 | C30H34O17 | 667.19 | -0.56 | 361.0912; 345.0611; 103.0388 | pos |
|  |  | 23.91 | C30H34O17 | 665.17 | 3.66 | 359.0753; 343.0444 | pos |
|  | Aerugidiol or its isomer (b) | 23.95 | C15H22O3 | 251.16 | 1.9 | 135.0803; 69.0698 | pos |
|  | Kaempferol-7-O-rhamnoside/Luteolin-7-O-rhamnoside/Kaempferol-3-O-rhamnoside (b) | 23.99 | C21H20O10 | 431.1 | -2.16 | 285.0387; 151.0019 | pos |
|  |  | 24.06 | C21H20O10 | 433.11 | 0.82 | 287.0544 | pos |
|  | Calycosin | 24.2 | C16H12O5 | 283.06 | 0.12 | 268.0374; 239.0340; 211.0389 | neg |
|  | Gancaonin B or its isomer (a) | 24.55 | C21H20O6 | 369.13 | 0.53 | 313.0683 | pos |
|  | Gomisin R or its isomer (a) | 24.84 | C22H24O7 | 401.16 | 1.44 | 383.1471; 315.0854 | pos |
|  | Luteolin or its isomer (c) | 24.85 | C15H10O6 | 285.04 | -2.82 | 151.0020; 133.0291; 107.0120 | pos |
|  | Suchengbeisine | 24.88 | C27H43NO3 | 430.33 | 1.13 | 412.3199 | pos |
|  | Luteolin or its isomer (c) | 24.9 | C15H10O6 | 287.05 | 0.67 | 241.0490; 153.0179 | pos |
|  | Liquiritigenin/Isoliquiritigenin or their isomer (c) | 24.91 | C15H12O4 | 255.07 | 0.59 | 119.0485; 135.0071 | neg |
|  | Icaritin-3-O-rhamnoside | 24.93 | C27H32O11 | 531.19 | -7.56 | 385.1266 | neg |
|  | Skimmetin or its isomer (b) | 24.97 | C9H6O3 | 163.04 | 0.01 | 135.0439 | pos |
|  | Pseudoginsenoside F11 or its isomer (a) | 25.18 | C42H72O14 | 799.48 | 9.28 | 359.3401 | neg |
|  | Licorice glycoside B/Licorice glycoside D1/Licorice glycoside D2 (b) | 25.85 | C35H36O15 | 695.2 | -2.23 | 549.1627; 531.1506; 285.0757; 255.0653 | neg |
|  | Gancaonin B or its isomer (b) | 25.9 | C21H20O6 | 369.13 | 5.74 | 313.0713 | pos |
|  | Melitidin | 26 | C33H40O18 | 725.23 | 7.16 | 419.1327; 404.1091; 361.0910 | pos |
|  | Gomisin R or its isomer (b) | 26.29 | C22H24O7 | 401.16 | 9.04 | 383.1487; 315.0858 | pos |
|  | Benzoylpaeoniflorin or its isomer (c) | 26.66 | C30H32O12 | 585.2 | 9.73 | 179.0699; 151.0751; 105.0333 | pos |
|  |  | 26.71 | C30H32O12 | 583.18 | 9.52 | 121.0277 | pos |
|  | Benzylidenemalonaldehyde | 26.72 | C10H8O2 | 161.06 | 9.86 | 133.0646; 105.0696 | pos |
|  | Epimedoside C | 27.1 | C26H28O11 | 517.17 | 9.72 | 355.1168; 299.0541 | pos |
|  |  | 27.11 | C26H28O11 | 515.16 | 3.46 | 353.1013; 297.0588 | pos |
|  | 20(S)-Ginsenoside Rg3/Ginsenoside F2/20(S)-Ginsenoside Rg3 (a) | 27.5 | C42H72O13 | 785.5 | 9.12 | 749.4739; 587.4205; 443.3852; 425.3781; 407.3665 | pos |
|  | Ginsenoside Rk2/Ginsenoside Rh3 or their isomer(a) | 27.72 | C36H60O7 | 605.44 | -8.65 | 189.1630; 147.1172; 133.1015; 107.0857; 95.0855; 69.0702 | pos |
|  | Gomisin H/Gomisin S (a) | 27.8 | C23H30O7 | 419.21 | 7.5 | 401.1962; 369.1681 | pos |
|  | Isolicoflavonol or its isomer (a) | 27.82 | C20H18O6 | 355.12 | 6.97 | 299.0561 | pos |
|  | Fritillarizine/Puqiedinone/Eduardine/Zhebeirine | 27.84 | C27H43NO2 | 414.34 | 8.43 | 396.3233 | pos |
|  | Paeonol | 27.88 | C9H10O3 | 167.07 | 7.85 | 149.0594; 121.0645; 91.0542 | pos |
|  | Ginsenoside Rh4/Rk3 | 27.92 | C36H60O8 | 621.44 | 8.02 | 441.3715; 423.3548; 405.3510 | pos |
|  | 20(S)-Ginsenoside Rh1 | 28.01 | C36H62O9 | 639.45 | 7.61 | 441.3721; 423.3610; 305.3492 | pos |
|  | Epimedoside A/Ikarisoside B or their isomer (c) | 28.16 | C32H38O15 | 661.21 | 9.82 | 353.0977 | neg |
|  | Desmethylanhydroicaritin or its isomer (a) | 28.17 | C20H18O6 | 355.12 | 6.97 | 299.0542 | pos |
|  | Apigenin or its isomer (c) | 28.77 | C15H10O5 | 269.04 | 1.85 | 151.0023; 117.0330 | neg |
|  | Ebeiedine/Puqiedine/N-demethylpuqietinone/Eduardinine | 29.02 | C27H45NO2 | 416.35 | 4.27 | 398.3392 | pos |
|  | 5-Hydroxy-7,8,4'-Trimethoxyflavanone or its isomer (b) | 29.02 | C18H16O6 | 329.1 | 3.33 | 314.0780; 299.0564 | pos |
|  | Maohuoside A | 29.08 | C27H32O12 | 549.2 | 4.75 | 387.1431; 369.1324; 313.0699 | pos |
|  |  | 29.09 | C27H32O12 | 547.18 | -5.08 | 385.1281 | pos |
|  | Epimedokoreanoside II/Sagittatoside C | 29.15 | C35H42O16 | 719.25 | 4.03 | 369.1322; 313.0696 | pos |
|  | Apigenin or its isomer (d) | 29.22 |  | 269.05 | -0.38 |  | pos |
|  | 5-Hydroxy-6,7,8,3',4'-Pentamethoxyflavone | 29.25 | C20H20O8 | 389.12 | 4.66 | 359.0753 | pos |
|  | Apigenin or its isomer (d) | 29.25 |  | 271.06 | 3.99 |  | pos |
|  | Monohydroxy-pentamethoxyflavone or its isomer (a) | 29.31 | C20H20O8 | 389.12 | 4.66 | 374.0999; 359.0749 | pos |
|  | (-)-Gomisin L2/(-)-Gomisin L1 (b) | 29.43 | C22H26O6 | 387.18 | 3.01 | 371.1576 | pos |
|  | Chrysoeriol or its isomer (c) | 29.99 | C16H12O6 | 299.06 | -0.55 | 284.0315; 256.0356; 239.0353 | pos |
|  |  | 30.12 | C16H12O6 | 301.07 | 1.35 | 286.0465; 258.0517 | pos |
|  | Desmethylanhydroicaritin or its isomer (b) | 30.16 | C20H18O6 | 355.12 | 3.45 | 299.0541 | pos |
|  | Ikarisoside F | 30.18 | C31H36O14 | 633.22 | 3.78 | 355.1172; 299.0543 | pos |
|  | 20(R)-Ginsenoside Rh1 or its isomer (b) | 30.28 | C36H62O9 | 639.45 | 2.46 | 441.3689; 423.3607 | pos |
|  | Kaempferol | 30.31 | C15H10O6 | 285.04 | -2.5 | 229.0495; 159.0436; 151.0020 | pos |
|  | Licoricesaponin A3 or its isomer (b) | 30.52 | C48H72O21 | 983.45 | 8.53 | 821.3954; 803.3823; 645.3605; 351.0565; 289.0556 | neg |
|  | Monohydroxy-pentamethoxyflavone or its isomer (b) | 30.7 | C20H20O8 | 389.12 | 1.05 | 374.0992; 359.0755 | pos |
|  | Hesperetin or its isomer (e) | 30.74 | C16H14O6 | 303.09 | 1.23 | 177.0542; 153.0179; 145.0281 | pos |
|  |  | 30.75 | C16H14O6 | 301.07 | 5.78 | 164.0098; 151.0019; 136.0147 | pos |
|  | Yunganoside G2 | 31.41 | C42H64O17 | 839.41 | 8.28 | 821.4069; 351.0561; 289.0561 | neg |
|  | Wyerone or its isomer (b) | 31.41 | C15H14O4 | 257.08 | -1.73 | 151.0384 | neg |
|  | Baohuoside II | 32.31 | C26H28O10 | 501.17 | 0.86 | 355.1168; 299.0542 | pos |
|  |  | 32.32 | C26H28O10 | 499.16 | -7.37 | 353.1014; 297.0396 | pos |
|  | Desmethylanhydroicaritin or its isomer (c) | 32.33 | C20H18O6 | 355.12 | 0.7 | 299.054 | pos |
|  | Licoricesaponin D3 or its isomer (a) | 32.44 | C50H76O21 | 1011.48 | 9.16 | 497.1123; 321.0820 | neg |
|  | Monohydroxy-pentamethoxyflavone or its isomer (c) | 32.56 | C20H20O8 | 389.12 | 0.19 | 359.0769; 341.0654 | pos |
|  | beta-Turmerone or its isomer (b) | 32.7 | C15H22O | 219.17 | -0.2 | 203.1432; 119.0853 | pos |
|  | Isomeramazin/Meranzin (b) | 33.1 | C15H16O4 | 261.11 | -0.38 | 243.1016; 189.0543; 131.0489 | pos |
|  | Glycyrrhetic acid isomer (a) | 33.43 | C30H46O4 | 471.35 | 1.41 | 317.2098; 235.1683; 189.1639 | pos |
|  | Glycyrrhizic acid | 33.44 | C42H62O16 | 821.4 | 9.9 | 759.3407; 645.3630; 351.0560 | neg |
|  | Liquiritigenin/Isoliquiritigenin or their isomer (d) | 33.44 | C15H12O4 | 255.07 | -3.08 | 135.0070; 119.0484 | neg |
|  | (3R)-Vestitol or its isomer (b) | 34.01 | C16H16O4 | 273.11 | 4.9 | 163.0751; 137.0592; 123.0437 | pos |
|  | Sinensetin or their isomer (a) | 34.01 | C20H20O7 | 373.13 | 3.02 | 343.0803; 315.0854 | pos |
|  | Uralsaponin C/Licorice saponin J2 or their isomer (b) | 34.2 | C42H64O16 | 823.41 | 9.91 | 351.0566 | neg |
|  | 20(S)-Ginsenoside Rg3/Ginsenoside F2/20(S)-Ginsenoside Rg3(b) | 34.96 | C42H72O13 | 785.5 | 9.43 | 425.3775; 407.3655 | pos |
|  | Protopanaxadiol/Panaxadiol | 34.98 | C30H52O3 | 461.4 | 9.23 | 443.3860; 425.3772 | pos |
|  | Myricadiol or its isomer (a) | 34.98 | C30H50O2 | 443.39 | 8.57 | 425.3765; 407.3673; 189.1638 | pos |
|  | Sagittatoside B | 36.35 | C32H38O14 | 647.23 | -0.5 | 369.1324; 313.0698 | pos |
|  | 2″-O-Rhamnosyl icariside II or its isomer (b) | 37.48 | C33H40O14 | 659.24 | 5.75 | 351.0866; 323.0911 | pos |
|  | Myricadiol or its isomer (b) | 40.97 | C30H50O2 | 443.39 | 9.19 | 425.3764; 407.3667; 189.1634 | pos |
|  | Ginsenoside Rg5/Rk1 or their isomer (a) | 41.09 | C42H70O12 | 767.49 | 8.8 | 425.3771; 407.3667 | pos |
|  | Gomisin J | 41.15 | C22H28O6 | 389.2 | 9.41 | 357.1685 | pos |
|  | Licoflavone A | 41.31 | C20H18O4 | 321.11 | 9.76 | 265.0464 | neg |
|  | 20(S)-Ginsenoside Rg3/Ginsenoside F2/20(S)-Ginsenoside Rg3 (c) | 41.36 | C42H72O13 | 785.5 | 8.27 | 425.3769; 407.3669 | pos |
|  | Icaritin/Gancaonin B or their isomer (f) | 41.46 | C21H20O6 | 369.13 | 9.71 | 313.0699; 285.0749; 270.0517 | pos |
|  | 5,7,4'-Trimethoxyflavone | 41.6 | C18H16O5 | 313.11 | 8.38 | 298.0827; 269.0799 | pos |
|  | Licochalcone B or its isomer (c) | 41.7 | C16H14O5 | 285.08 | 5.63 | 243.0655 | neg |
|  | Desmethylanhydroicaritin or its isomer (d) | 42.25 | C20H18O6 | 355.12 | 6.72 | 299.0546 | pos |
|  | Schizandrol B or its isomer (b) | 42.39 | C23H28O7 | 417.19 | 8 | 399.1792; 369.1683; 299.1250 | pos |
|  | Tangeretin | 43.03 | C20H20O7 | 373.13 | 4.41 | 343.0804; 315.0848; 297.0750 | pos |
|  | Desmethylanhydroicaritin or its isomer (e) | 43.71 | C20H18O6 | 353.1 | 1.67 | 297.0398 | pos |
|  |  | 43.72 | C20H18O6 | 355.12 | 3.71 | 299.0538 | pos |
|  | Ginsenoside Rs4/Ginsenoside Rs5 | 43.99 | C44H72O13 | 809.5 | 2.23 | 407.3639 | pos |
|  | Nomilin | 43.99 | C28H34O9 | 515.23 | 2.94 | 369.1639; 307.0953; 161.0598 | pos |
|  | 20(S)-Ginsenoside Rs3/20(R)-Ginsenoside Rs3 | 44.01 | C44H74O14 | 827.51 | 3.56 | 407.3686 | pos |
|  | Emodin | 44.57 | C15H10O5 | 269.05 | -1.75 | 241.0498; 225.0544 | neg |
|  | Schisandrin B isomer (a) | 44.65 | C23H28O6 | 401.2 | 3.47 | 301.1065; 300.0963; 285.0752; 227.0699 | pos |
|  | Tigloylgomisin H/Angeloylgomisin H (a) | 44.73 | C28H36O8 | 501.25 | 3.77 | 483.2371; 401.1950 | pos |
|  | Ginsenoside Rg5/Ginsenoside Rk1 or their isomer (b) | 45.2 | C42H70O12 | 767.49 | 10 | 605.4400; 443.3867; 425.3765; 407.3668 | pos |
|  | Ginsenoside Rk2/Ginsenoside Rh3 or their isomer (c) | 45.22 | C36H60O7 | 605.44 | 9.09 | 425.3806 | pos |
|  | Myricadiol or its isomer (c) | 45.22 | C30H50O2 | 443.39 | 9.39 | 425.3767;407.3637; 189.1633 | pos |
|  | Epigomisin O | 45.34 | C23H28O7 | 417.19 | 8 | 399.1802; 369.1671 | pos |
|  | Icaritin/Gancaonin B or their isomer (g) | 45.51 | C21H20O6 | 369.13 | 9.71 | 313.0709 | pos |
|  | Desmethylanhydroicaritin or its isomer (f) | 45.53 | C20H18O6 | 355.12 | 9.81 | 299.0544 | pos |
|  | Monohydroxy-pentamethoxyflavone or its isomer (d) | 45.55 | C20H20O8 | 389.12 | 9.28 | 359.0741 | pos |
